# Neutrophil recruitment limited by high-affinity bent β_2_ integrin binding ligand in *cis*

**DOI:** 10.1038/ncomms12658

**Published:** 2016-08-31

**Authors:** Zhichao Fan, Sara McArdle, Alex Marki, Zbigniew Mikulski, Edgar Gutierrez, Britta Engelhardt, Urban Deutsch, Mark Ginsberg, Alex Groisman, Klaus Ley

**Affiliations:** 1 Division of Inflammation Biology, La Jolla Institute for Allergy and Immunology, 9420 Athena Circle Drive, La Jolla, California 92037, USA; 2 Department of Bioengineering, University of California San Diego, 9500 Gilman Drive, La Jolla, California 92093, USA; 3 Department of Physics, University of California San Diego, 9500 Gilman Drive, La Jolla, California 92093, USA; 4 Theodor Kocher Institute, University of Bern, 1 Freiestrasse, 3012 Bern, Switzerland; 5 Department of Medicine, University of California San Diego, 9500 Gilman Drive, La Jolla, California 92093, USA

## Abstract

Neutrophils are essential for innate immunity and inflammation and many neutrophil functions are β_2_ integrin-dependent. Integrins can extend (E^+^) and acquire a high-affinity conformation with an ‘open' headpiece (H^+^). The canonical switchblade model of integrin activation proposes that the E^+^ conformation precedes H^+^, and the two are believed to be structurally linked. Here we show, using high-resolution quantitative dynamic footprinting (qDF) microscopy combined with a homogenous conformation-reporter binding assay in a microfluidic device, that a substantial fraction of β_2_ integrins on human neutrophils acquire an unexpected E^−^H^+^ conformation. E^−^H^+^ β_2_ integrins bind intercellular adhesion molecules (ICAMs) in *cis*, which inhibits leukocyte adhesion *in vitro* and *in vivo*. This endogenous anti-inflammatory mechanism inhibits neutrophil aggregation, accumulation and inflammation.

## Abstract (web summary)

Integrin β_2_ attachment regulates inflammation via effects on neutrophil rolling and extravasation through sequential integrin extension then headpiece opening. Here the authors show an alternative open headpiece prior to extension stabilized in *cis* by ICAM-1 that limits neutrophil adhesion.

## Graphical abstract

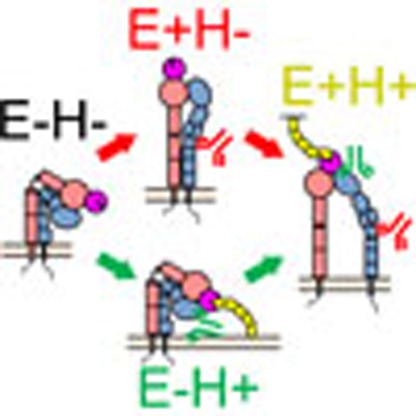

Neutrophils are the most abundant leukocyte population in humans and have essential roles in innate immunity and inflammation. Neutrophils form a primary line of defence against pathogens by recognizing, trapping and eliminating bacteria^1^, fungae^2^, viruses^3^ and helminths^4^ through mechanisms including phagocytosis^5^, superoxide production^5^, granule exocytosis^6^ and neutrophil extracellular traps^1^. Congenital deficiencies of neutrophil function, such as leukocyte adhesion deficiency syndrome, can result in fatal infection^7^. Delay in neutrophil recovery is the main reason for lethal infections in patients receiving allogeneic haematopoietic stem cell transplantation^8^. Neutrophils are also important in non-infectious or chronic inflammation such as injury-induced sterile inflammation^9^, and in autoimmune diseases, including rheumatoid arthritis^10^, multiple sclerosis^11^ and systemic lupus erythematosus^12^.

Neutrophils are distributed by the blood circulation, but exert most of their functions outside the vascular system, exiting the circulation at sites of inflammation through the classical adhesion cascade^13^,^14^. Neutrophils have evolved specialized systems to allow adhesion under high wall shear stress^13^,^15^,^16^. β_2_ integrins are vital for neutrophil functions, including neutrophil arrest under flow^13^, transmigration through the vascular endothelium^17^, chemotaxis^18^ and phagocytosis^19^.

The leukocyte adhesion cascade was first established when Lawrence and Springer showed that neutrophils rolling on P-selectin were induced to arrest under flow by binding to intercellular adhesion molecule-1 ((ICAM-1), a ligand for β_2_ integrins) when the chemoattractant *N*-formylmethionine-leucyl-phenylalanine was infused^14^. Similar results were shown for neutrophil rolling and arrest *in vivo*^20^ and in flow chamber studies in which the chemokine interleukin (IL)-8 was co-immobilized with ICAM-1 and P-selectin^21^. Rolling neutrophils arrest by β_2_ integrin^22^ activation through inside-out signalling^23^, which is thought to be rapid and local^24^; however, the subcellular distribution of integrin activation is unknown.

Integrins are αβ heterodimers that regulate their adhesiveness through changes in the conformation of their ectodomain^25^,^26^. Resting integrins assume a bent conformation with low affinity for ligand. The ‘switchblade' model of integrin activation (Supplementary Fig. 1a)^25^ suggests a two-step activation process in which integrin extension (E^+^) is followed by a rearrangement in the ligand-binding site leading to high-affinity (H^+^). β_2_ integrin extension is detected by monoclonal antibody (mAb) KIM127, which recognizes a neoepitope^27^ that is hidden in the bent knee of human β_2_ (ref. 28). On the other hand, when human β_2_ integrins acquire high affinity, a neoepitope in the β_2_ I-like domain^29^,^30^,^31^ is exposed, which is recognized by mAb24 (ref. 32). Thus, KIM127 binding indicates E^+^ and mAb24 binding indicates H^+^. KIM127 and mAb24 do not block each other and do not block ligand binding.

Research has shown that P-selectin glycoprotein ligand-1 (PSGL-1) signalling triggers integrin extension^33^,^34^. This signalling cascade starts with L-selectin and PSGL-1 (ref. 35), proceeds through various signalling intermediates^26^,^34^ and induces the E^+^ but not the H^+^ integrin conformation^33^,^34^. Another signalling cascade is triggered when a chemokine binds to its cognate G-protein-coupled receptor^26^. This binding induces dissociation of Gαi2 from Gβγ, and is required for arrest^36^. A distal signalling cassette involving Rap-1, Rho^37^, Rap1-GTP-interacting adaptor molecule^38^,^39^, talin^40^,^41^ and kindlin-3 (refs 7, 41) has been described. G-protein-coupled receptor signalling is thought to trigger H^+^ or possibly both E^+^ and H^+^ (ref. 23).

Quantitative dynamic footprinting (qDF) microscopy, which is based on variable angle total internal reflection fluorescence (TIRF) to provide nanometer resolution in the vertical *z* axis, was combined with microfluidics^15^,^16^ to study neutrophil interaction with defined molecular substrates at the subcellular level. This method provides precise maps of the neutrophil surface and the location of relevant molecules. Here we modify and expand qDF microscopy to three colours and combine it with a homogeneous binding assay^33^, which introduces soluble, fluorescence-labelled mAb24 and KIM127, to investigate the dynamics of β_2_ integrin activation during primary human neutrophil rolling and arrest under physiologic conditions. We find an unexpected E^−^H^+^ conformation of β_2_ integrins that interacts with ICAMs in *cis*. This *cis* interaction inhibits leukocyte adhesion and aggregation, thus providing an auto-inhibitory mechanism that curbs inflammatory responses.

## Results

### β_2_ integrin activation on rolling human neutrophils

**Figure 1:**
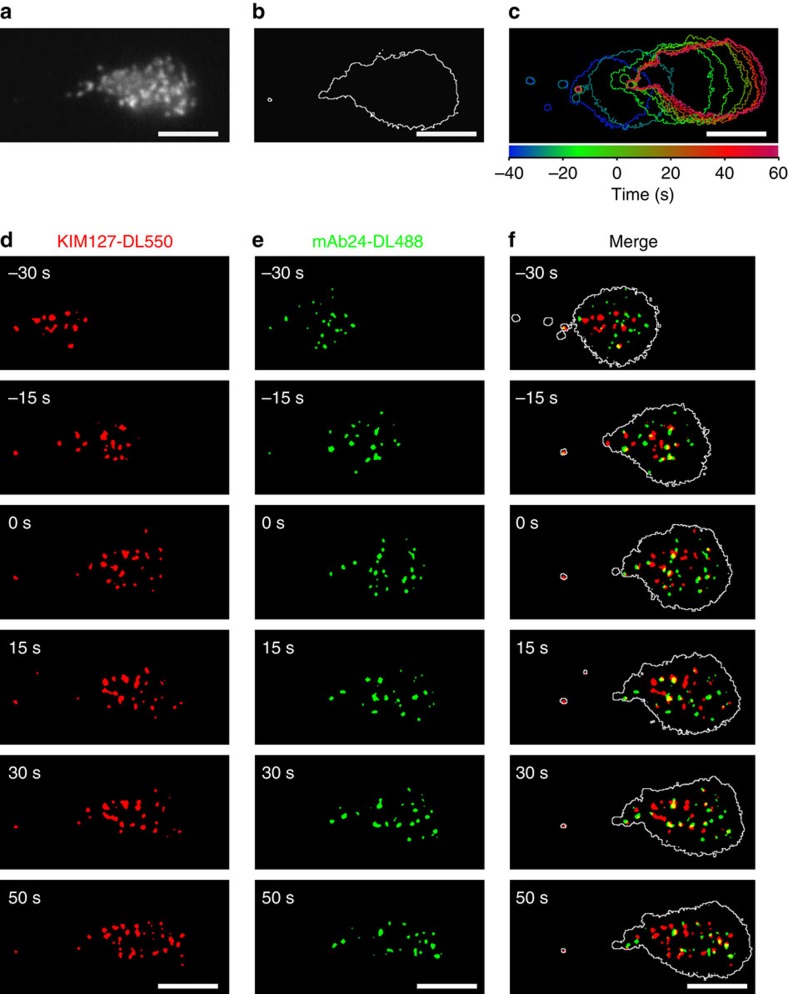
β_2_ integrin E^+^ and H^+^ conformations on human neutrophil footprint. (**a**) A typical image of fluorescently labelled neutrophil membrane. (**b**) Footprint outline of a neutrophil generated from membrane fluorescence in **a**. (**c**) Footprint outlines of a typical cell during rolling on the P-selectin/ICAM-1/IL-8 substrate at a wall shear stress of 6 dyn cm^−2^ (arrest at time=0 s); time was coded in rainbow colours as shown in colour bar. (**d**–**f**) E^+^ β_2_ integrins identified by KIM127-DL550 (red in **d** and **f**), and H^+^ by mAb24-DL488 (green in **e** and **f**) during neutrophil rolling on P-selectin/ICAM-1/IL-8 substrate. Footprint outlines shown in white in **f**. Binary images generated by smart segmentation; E^+^H^+^, E^+^H^−^ and E^−^H^+^ clusters appear yellow, red and green, respectively, in **f**; scale bars, 5 μm.

**Figure 2:**
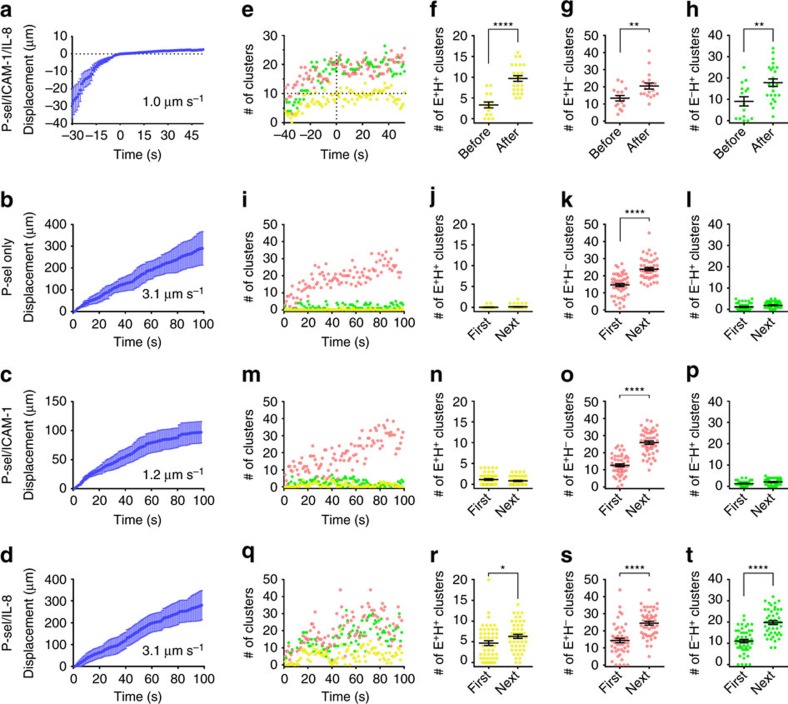
Differential effects of ICAM-1 and IL-8 on integrin activation in human neutrophils. (**a**–**d**) Displacements of typical cells (*n*=9, mean±s.e.m.) during rolling on P-selectin/ICAM-1/IL-8 (**a**, arrest at time=0 s), P-selectin only (**b**), P-selectin/ICAM-1 (**c**) or P-selectin/IL-8 (**d**) substrates, respectively. Average rolling velocity (for **a**: before arrest) determined from linear regression. (**e**) Dynamics of cluster number per cell (E^+^H^−^ red, E^−^H^+^ green, E^+^H^+^ yellow) rolling on P-selectin/ICAM-1/IL-8. (**f**–**h**) Number of E^+^H^+^ (**f**), E^+^H^−^ (**g**) or E^−^H^+^ (**h**) clusters averaged before (−30 and −15 s, *n*=16) and after (0, 15 and 30 s, *n*=24) arrest in eight cells rolling on P-selectin/ICAM-1/IL-8; each time point of each cell represented by one dot, mean±s.e.m. **P<0.01, **** *P*<0.0001. (**i**–**t**) E^+^H^−^ (red), E^−^H^+^ (green) and E^+^H^+^ (yellow) clusters for neutrophils rolling on P-selectin only (**i**), P-selectin/ICAM-1 (**m**), and P-selectin/IL-8 (**q**) coated substrates. E^+^H^+^ (**j**,**n**,**r**), E^+^H^−^ (**k**,**o**,**s**) and E^−^H^+^ (**l**,**p**,**t**) clusters in the footprint of cells rolling on P-selectin only (**j**–**l**), P-selectin/ICAM-1 (**n**–**p**) or P-selectin/IL-8 (**r**–**t**) in the first 50 s (first) and the next ∼50 s (next) of rolling. Mean values and s.e.ms (error bars) were presented. * *P*<0.05, **** *P*<0.0001 in unpaired student *t*-test.

Microfluidic chambers^15^ were coated with recombinant human P-selectin-Fc (to support rolling), ICAM-1-Fc (a ligand for both α_L_β_2_, lymphocyte function-associated antigen (LFA)-1 and α_M_β_2_, macrophage-1 antigen, Mac-1) and IL-8 with all concentrations titrated so that neutrophils would arrest only when all three molecules were present (Supplementary Fig. 2a). Both LFA-1 and Mac-1 contributed to human neutrophil arrest (Supplementary Fig. 2a). Soluble KIM127 and mAb24 did not shown any significant influence on neutrophil rolling and arrest (Supplementary Fig. 2b–e). Both antibodies bound rapidly (within one video frame) to immobilized activated neutrophils (Supplementary Fig. 3) with no evidence for the loss of binding over time, underscoring the validity of the homogeneous binding assay. Neutrophils isolated from anticoagulated blood and labelled with membrane dye (CellMask DeepRed) were perfused in the presence of DyLight 550 (DL550) conjugated KIM127 and DyLight 488 (DL488) conjugated mAb24 at 6 dyn cm^−2^ and imaged with a newly developed triple-colour qDF (TqDF) setup. Smart segmentation image processing (Supplementary Fig. 4) was used to remove background and generate binary images of the neutrophil footprint in contact with the substrate (Fig. 1a,b) from raw images (Supplementary Fig. 5a) and reproduce the cluster morphology with better signal-to-noise ratio (Supplementary Fig. 5b). On the P-selectin/ICAM-1/IL-8 substrate, neutrophils rolled and arrested (Fig. 1c). Unlike the nearly homogeneous distribution of total α_L_β_2_ integrins on the cell surface, both KIM127^+^ and mAb24^+^ β_2_ integrins were present in small clusters (Fig. 1d–f, Supplementary Figs 4 and 5 and Supplementary Movie 1) before arrest (time=0 s) and remained in clusters of similar size (Supplementary Fig. 6) after arrest. In the overlaid images (Fig. 1f and Supplementary Movie 1), E^+^H^−^ (KIM127^+^mAb24^−^, red) and E^+^H^+^ (KIM127^+^mAb24^+^, yellow) β_2_ integrins were observed during neutrophil rolling and arrest as expected. Unexpectedly, neutrophils also showed clusters of mAb24^+^KIM127^−^ β_2_ integrins (E^−^H^+^, green). Very few clusters of mAb24^+^KIM127^+^ integrins (E^+^H^+^, yellow, time before arrest) are observed in rolling neutrophils before arrest. Dye switch experiments excluded nonspecific effects of the fluorochromes used (Supplementary Fig. 7a). These experiments show that neutrophils rolling on ‘complete' substrate (P-selectin/ICAM-1/IL-8) show the complete physiologic transition from rolling to arrest within ∼30 s (Fig. 2a) and express small (∼0.1 μm^2^) clusters of E^+^H^−^, E^−^H^+^ and E^+^H^+^ β_2_ integrins.

### Different roles of P-selectin and IL-8

To assess which component on the substrate induces integrin activation, we tested neutrophil rolling and adhesion on ‘incomplete' substrates: P-selectin only, P-selectin/ICAM-1 and P-selectin/IL-8 (Fig. 2). On the ‘complete' P-selectin/ICAM-1/IL-8 substrate, neutrophils rolled at a velocity of ∼1.0 μm s^−1^ (Fig. 2a) before arrest at time=0. As expected^34^, neutrophils rolled much faster (∼3.1 μm s^−1^) on P-selectin only (Fig. 2b), whereas the P-selectin/ICAM-1 substrate (Fig. 2c) supported slow rolling (∼1.2 μm s^−1^), but no arrest. Adding IL-8 to the P-selectin substrate (Fig. 2d) did not reduce rolling velocity (∼3.1 μm s^−1^) and did not support arrest. Quantitative analysis of the cluster number (Fig. 2e) showed that neutrophils rolling on P-selectin/ICAM-1/IL-8 substrate started with ∼9 E^+^H^−^, ∼9 E^−^H^+^ and ∼3 E^+^H^+^ clusters at −30 s. As the cells continued rolling, the number of E^+^H^+^ clusters increased and reached 9±1 when the cells arrested (time=0 s, Fig. 2e and Supplementary Fig. 6a). The step change from pre-arrest to arrest was highly significant (Fig. 2f). The number of E^+^H^−^ clusters (Fig. 2g) and E^−^H^+^ clusters (Fig. 2h) also significantly increased upon arrest. The total area of E^+^H^−^, E^−^H^+^ and E^+^H^+^ clusters increased in proportion to the cluster number (Supplementary Fig. 6b) and the size of each cluster did not change significantly (Supplementary Fig. 6c).

When neutrophils were rolling on P-selectin only (Fig. 2i–l), E^+^H^−^ clusters were induced (red, Fig. 2i,k), as expected^33^,^34^, but no E^+^H^+^ clusters (yellow, Fig. 2i,j) or E^−^H^+^ clusters (green, Fig. 2i,l) were observed. Induction of E^+^H^−^ clusters but not E^−^H^+^ or E^+^H^+^ clusters was highly significant when comparing the first 50 s and the next ∼50 s of rolling (Fig. 2j–l). Rolling neutrophils on P-selectin/ICAM-1 substrate (no chemokine, Fig. 2m–p) produced a similar increase in E^+^H^−^ integrin (red, Fig. 2m,o) as on P-selectin. As expected, the cells rolled more slowly because the E^+^H^−^ integrin was able to bind to ICAM-1 with intermediate affinity. Neither E^+^H^+^ integrin (yellow, Fig. 2m,n) nor E^−^H^+^ integrin (green, Fig. 2m,p) were observed. This changed drastically when chemokine was available on the P-selectin/IL-8 substrate (no ICAM-1, Fig. 2q–t). Strikingly, E^+^H^+^ clusters (yellow, Fig. 2r) and E^−^H^+^ clusters (green, Fig. 2t) were induced along with the expected E^+^H^−^ clusters (red, Fig. 2s). Taken together, these data suggest that P-selectin binding is sufficient to induce integrin extension and chemokine is necessary to induce headpiece-opening.

### E^+^H^+^ clusters derived from both E^+^H^−^ and E^−^H^+^ clusters

**Figure 3:**
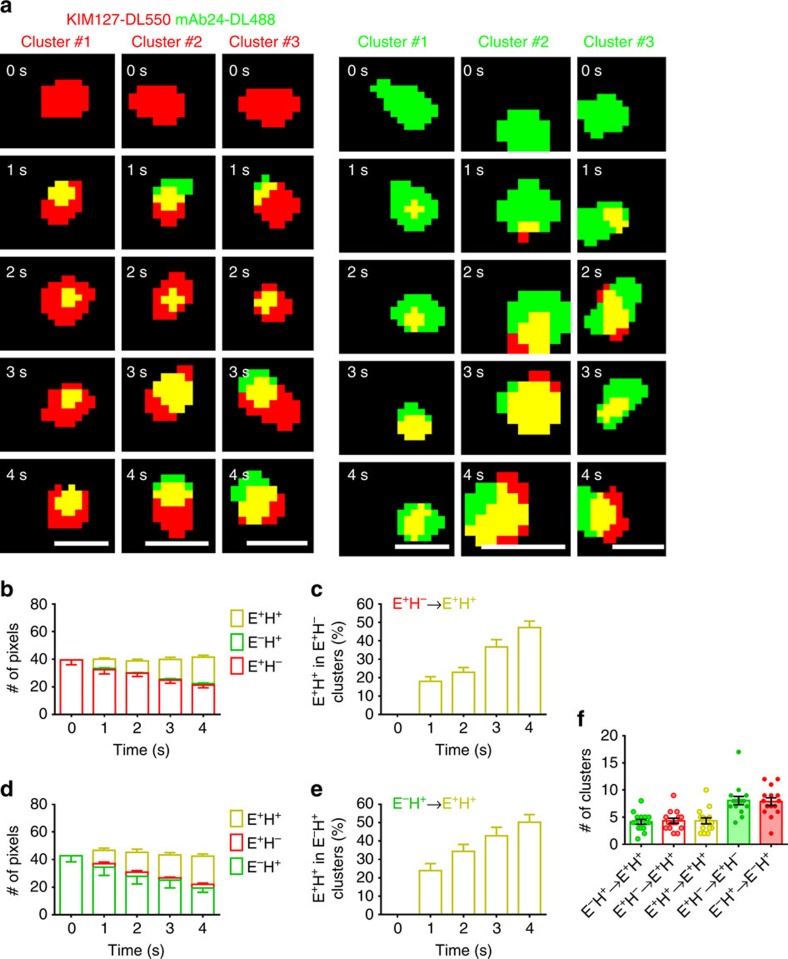
Two pathways of conformational transitions during β_2_ integrin activation. (**a**) Three examples of KIM127-DL550 (red, E^+^H^−^) or mAb24-DL488 (green, E^−^H^+^) clusters transitioning to E^+^H^+^ (yellow) over 4 s in the footprint of primary human neutrophils rolling on P-selectin, ICAM-1 and IL-8; scale bars, 0.5 μm. (**b**–**e**) Mean values of pixel numbers per cluster in colours as indicated (**b**,**d**) and percentage of E^+^H^+^ pixels (**c**,**e**) of 15 clusters starting as E^+^H^−^ (**b**,**c**) or 15 clusters starting as E^−^H^+^ (**d**,**e**); s.e.ms were shown as error bars. Data collected from static cells (pre-arrest and arrested). (**f**) Transition history of the clusters on arrested cells (*n*=15, one dot per cell). Mean values and s.e.ms (error bars) were presented.

The strong dependence of arrest on the appearance of ∼9 E^+^H^+^ clusters (Fig. 2e and Supplementary Fig. 6a) suggests that E^+^H^+^ integrins are the functional entity for binding ICAM-1 in trans. When focusing on individual clusters labelled with KIM127-DL550 or mAb24-DL488, we observed that both E^+^H^−^ integrins (red) and E^−^H^+^ integrins (green) transitioned to E^+^H^+^ (yellow, Fig. 3a). Dye switch experiments excluded nonspecific effects of the fluorochromes used (Supplementary Fig. 7b). About one-third of E^+^H^−^ clusters became E^+^H^+^ within 4 s (Fig. 3b,c, *n*=15). E^−^H^+^ clusters also became E^+^H^+^ at a similar rate (Fig. 3d,e, *n*=15). When tracking the history of the clusters on arrested cells, many E^−^H^+^ and E^+^H^−^ clusters remained E^−^H^+^ or E^+^H^−^, respectively, but some clusters (∼5 per neutrophil) converted from E^+^H^−^ or E^−^H^+^ to E^+^H^+^ (Fig. 3f). This was caused by an increase in the number of double positive (red and green) pixels over time and independent of small random fluctuations in background intensity (Supplementary Fig. 8). These findings suggest a new alternative pathway (Supplementary Fig. 1b) in which integrin undergoes a conformational change from E^−^H^−^ to E^−^H^+^ first and then to E^+^H^+^, clearly different from the canonical pathway suggested by the switchblade model. These two pathways both contributed to fully activated (E^+^H^+^) integrins on neutrophils during transition from rolling to arrest on P-selectin/ICAM-1/IL-8 substrate.

### Three-dimensional localization of integrin activation

**Figure 4:**
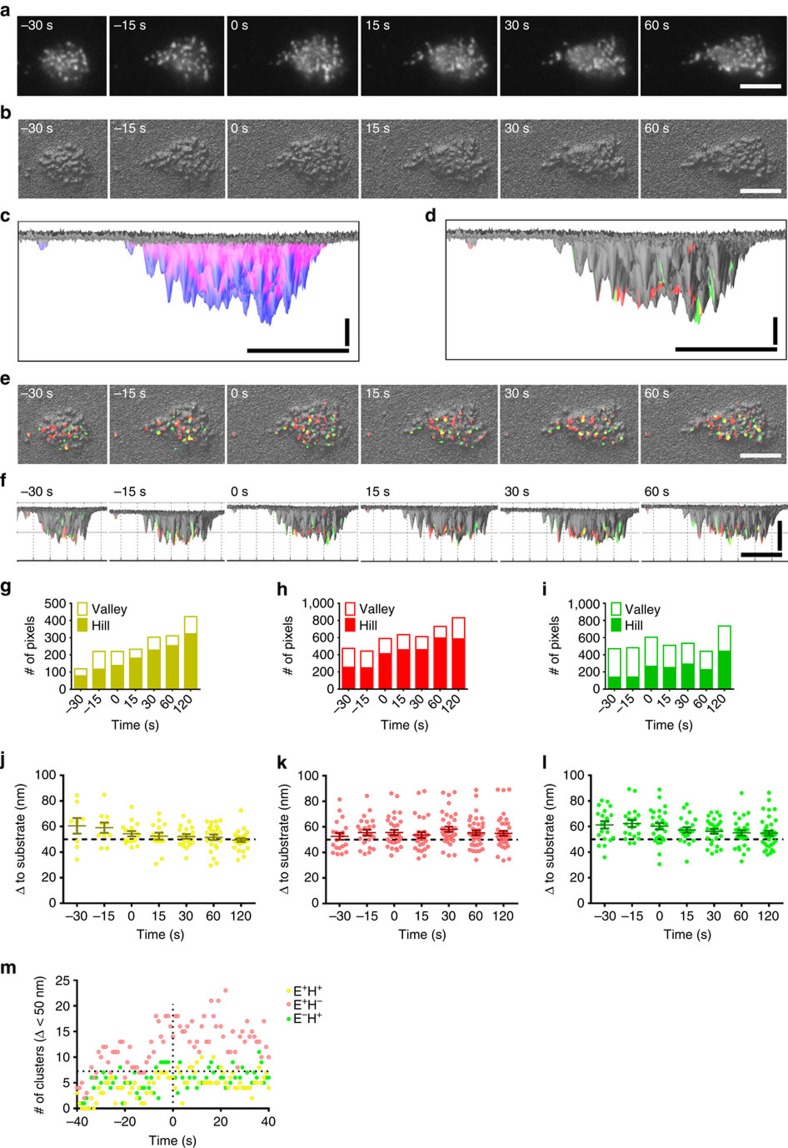
3D distributions of β_2_ integrin activation clusters in primary human neutrophils. (**a**) Neutrophil membrane (CellMask DeepRed) before and after arrest (0 s) of one representative neutrophil rolling on P-selectin, ICAM-1 and IL-8. (**b**) Membrane signal converted to hills (microvilli) and valleys (space between microvilli). (**c**,**d**) Hills (blue) and valleys (magenta) segmented (**c**) or E^+^H^−^ (red), E^−^H^+^ (green) or E^+^H^+^ (yellow) clusters (**d**) were identified at time of arrest (0 s). (**e**,**f**) Top-view (**e**) and side-view (**f**) of the 3D topography overlaid with E^+^H^−^ (red), E^−^H^+^ (green) or E^+^H^+^ (yellow) clusters; binary images. Horizontal scale bars, 5 μm, vertical scale bar, 20 nm for **d**, scale bar, 50 nm for **f**. (**g**–**i**) Most E^+^H^+^ (**g**, 70±4%) and E^+^H^−^ (**h**, 68±4%) cluster pixels were on hills. Most E^−^H^+^ cluster pixels (**i**, 71±0%) were in valleys before arrest (*P*<0.01 and *P*<0.05 compared with E^+^H^−^ and E^+^H^−^ cluster pixels in unpaired student *t*-test, respectively) and more E^−^H^+^ cluster pixels (52±6%) localized to the hills after arrest (*P*<0.05 and *P*<0.01 compared with E^+^H^−^ and E^+^H^−^ cluster pixels in unpaired student *t*-test, respectively). The E^+^H^+^ (**g**), E^+^H^−^ (**h**) and E^−^H^+^ (**i**) cluster pixels on the hills increased with time (the slopes were significantly non-zero, *F*-test, *P*<0.01). (**j**–**l**) Distance (Δ) of E^+^H^+^ (yellow, **j**), E^+^H^−^ (red, **k**) or E^−^H^+^ (green, **l**) integrin clusters to the substrate. The dashed line at 50 nm separates the integrin clusters within reach (≤50 nm) from those beyond reach (>50 nm). Each cluster represented by one dot, mean values and s.e.ms (error bars) were presented. (**m**) Number of clusters within 50 nm to the substrate per cell (E^+^H^−^ red, E^−^H^+^ green, E^+^H^+^ yellow) during rolling on the substrate of P-selectin/ICAM-1/IL-8 (arrest at 0 s).

E^+^H^+^ integrins can bind ligand in trans with high affinity. The E^+^H^+^ conformation is a necessary, but not sufficient condition for binding, since the ligand-binding I domain of α_L_ or α_M_ is only about 23 nm^42^ above the plasma membrane when extended. The extended β_2_ integrin-ICAM-1-assembly is about 42 nm long^43^. Neutrophils have microvilli that are ∼200 nm high^44^, and β_2_ integrins are known to be located both on microvilli (hills) and in the ‘valleys' between microvilli^45^. For E^+^H^+^ β_2_ integrins to reach ligand in trans, they effectively need to be near the top of the microvilli. To test what fraction of integrin clusters met these criteria, we converted^15^ the raw membrane data (Fig. 4a) into 3D footprints (Fig. 4b). Automated smart segmentation showed 27±1% hills and 73±1% valleys (Fig. 4c and Supplementary Fig. 9). Next, we superimposed E^+^H^+^ (yellow), E^+^H^−^ (red) and E^−^H^+^ (green) integrin clusters (Fig. 4d,e) on the 3D topography. Rotation by 90 degrees (Fig. 4d,f and Supplementary Movie 2) allowed us to map all clusters within ∼100 nm from the surface. Interestingly, significantly more (*P*<0.05) of the E^+^H^+^ (Fig. 4g, 70±4%) and E^+^H^−^ (Fig. 4h, 68±4%) clusters were on hills and thus close to the substrate than E^−^H^+^ clusters (Fig. 4i). The fraction of E^+^H^+^ and E^+^H^−^ integrin on hills significantly increased with time of rolling and continued to increase after arrest (time=0 s).

Integrin can bind ICAM-1 on the substrate only when the integrin is within ∼50 nm from the substrate (Supplementary Fig. 10). Analyzing the number of E^+^H^−^, E^+^H^+^ and E^−^H^+^ clusters within 50 nm of the substrate shows that during rolling, about three E^+^H^+^ clusters are ‘within reach', and the number of E^+^H^+^ clusters close to the substrate (Fig. 4j, yellow) continues to increase until arrest. The number of E^+^H^−^ clusters (Fig. 4k, red) within 50 nm of the substrate also increases during rolling. Some E^−^H^+^ clusters (Fig. 4l, green) are also within 50 nm, but this is irrelevant to ligand binding, because the bent conformation is not expected to bind ligand in trans even if the headpiece is ‘open' (Supplementary Fig. 10). The dynamics of integrin conformations within 50 nm to the substrate over time of rolling and arrest is shown in Fig. 4m, which shows that arrest is triggered by ∼7 E^+^H^+^ clusters that are close enough to the substrate to bind ICAM-1 in trans.

To directly measure the number of fully activated E^+^H^+^ β_2_ integrins that are needed for arrest, we measured the sites of KIM127 and mAb24 on the neutrophil surface by calibration beads with known antibody-binding sites using flow cytometry. Calculating from the fluorescence intensity integral of E^+^H^+^ clusters at the time of arrest (*t*=0), we find that 258±41 E^+^H^+^ integrin molecules are present in the neutrophil footprint at the time of arrest.

### E^−^H^+^ β_2_ integrins bind ICAM-1 on neutrophils in *cis*

**Figure 5:**
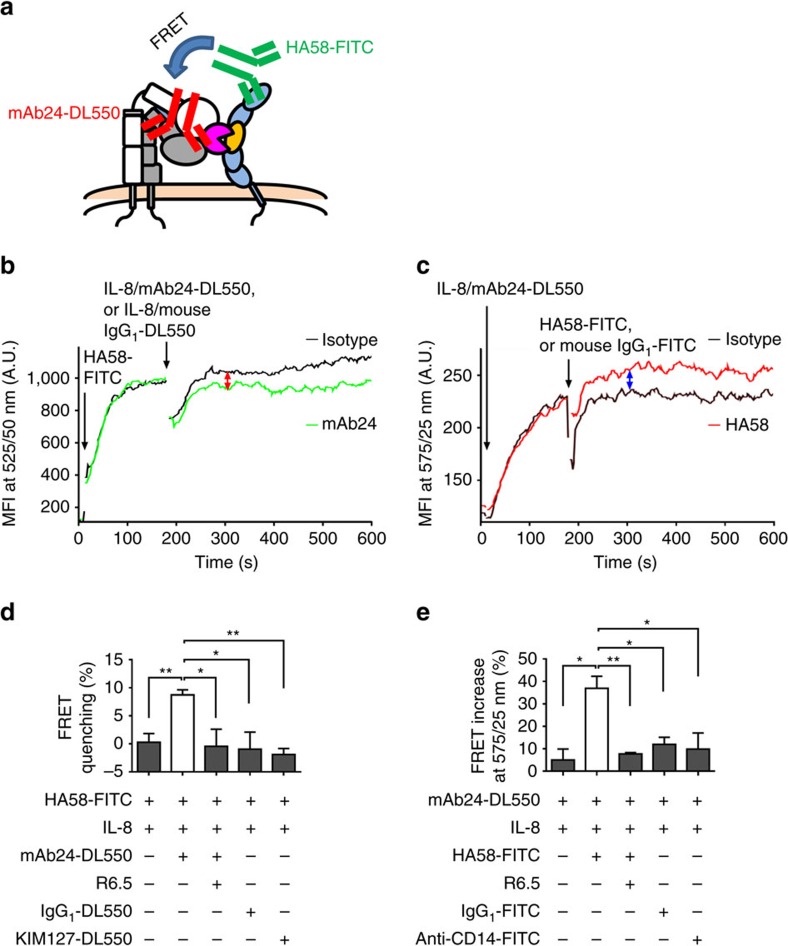
E^−^H^+^ Mac-1 binds ICAM-1 in *cis*. (**a**) Schematic of assessing the *cis* interaction between E^−^H^+^ Mac-1 and neutrophil ICAM-1 by FRET between ICAM-1 domain 1 (HA58-FITC, donor) and H^+^ integrin (mAb24-DL550, acceptor). (**b**,**c**) Donor fluorescence decrease (green in **b**) and acceptor fluorescence increase (red in **c**) shows FRET of HA58-FITC with mAb24-DL550, but not with isotype controls (IgG_1_-DL550 as acceptor, black in **b**; and IgG_1_-FITC as donor, black in **c**). (**d**,**e**) Donor fluorescence decrease (**d**) and acceptor fluorescence increase (**e**) of HA58-FITC-mAb24-DL550 pairs and controls measured at 2–3 min after adding IL-8 and acceptor or donor, respectively. Blocking of E^−^H^+^ Mac-1-ICAM-1 interactions (mAb R6.5) eliminated the donor fluorescence decrease and acceptor fluorescence increase. *n*=3, mean values and s.e.ms (error bars) were presented. * *P*<0.05, ** *P*<0.01 in unpaired student *t*-test. A.U., arbitrary unit.

The discovery of E^−^H^+^ β_2_ integrins on human neutrophils is the first report of E^−^H^+^ integrins on any physiological relevant cell. We reasoned that such bent high-affinity integrins may have a specific function. Since E^−^H^+^ integrin is not expected to bind ligand in trans, we considered whether E^−^H^+^ integrin may bind ligand in *cis*, that is, ICAM-1 expressed on the neutrophil. Human LFA-1 and Mac-1 bind domain 1 (ref. 46) and domain 3 (ref. 47) of human ICAM-1, respectively. To directly test whether E^−^H^+^ LFA-1 and Mac-1 could bind ICAM-1 in *cis* (on the neutrophil), we conducted Förster resonance energy transfer (FRET) experiments that report proximity of molecules within 1–10 nm (Fig. 5). When FRET occurs, emission at the shorter wavelength donor fluorochrome (for example, fluorescein isothiocyanate, FITC) is reduced (quenching at 525/50 nm), because some energy is transferred to the higher wavelength acceptor fluorochrome (for example, DL550). Conversely, FRET increases the emission of the higher wavelength fluorochrome (for example, DL550, measured at 575/25 nm). We reasoned that FRET should occur between mAb24 (binding β_2_ H^+^) and ICAM-1 domain 1 detected by mAb HA58 (Fig. 5a). Since mAb HA58 is function-blocking^48^ (disables ICAM-1 domain 1 binding to LFA-1), this assay directly tests the interaction of Mac-1 with domain 3 of ICAM-1. We indeed observed a significant decrease in donor fluorescence (Fig. 5b) and significant increase in acceptor fluorescence (Fig. 5c). This was specific, because FRET quenching did not occur when the acceptor mAb24-DL550 was absent or replaced by an isotype control antibody or KIM127-DL550, or when Mac-1 binding to ICAM-1 was blocked by mAb R6.5 (Fig. 5d)^49^. Similarly, the gain of acceptor fluorescence was blocked by adding R6.5, or when an irrelevant donor was used (anti-CD14-FITC, or isotype control, Fig. 5e) instead of HA58-FITC. Based on the FRET efficiency as measured, the average distance between the β_2_ I-like domain (mAb24-binding site) and ICAM-1 domain 1 (HA58-binding site) is calculated to be ∼9.9 nm, which is consistent with the known molecular dimensions.

**Figure 6:**
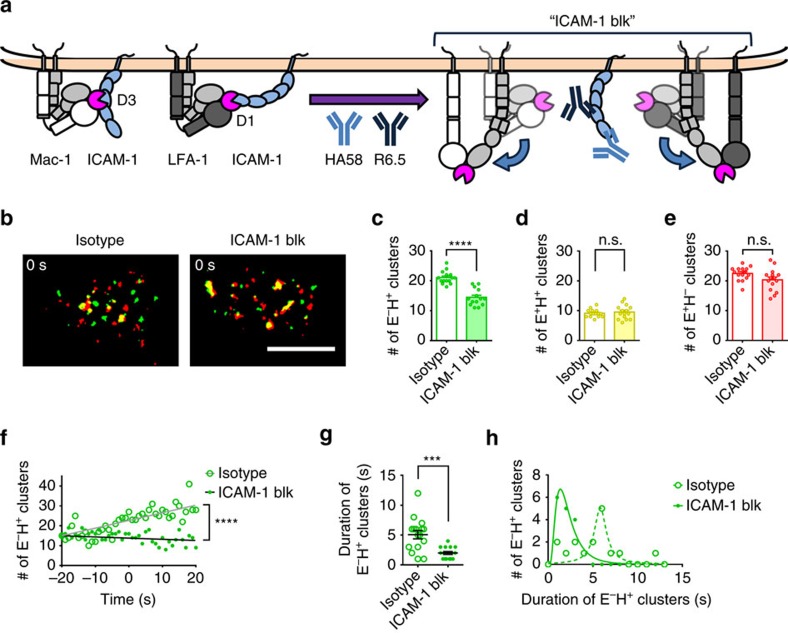
Blocking E^−^H^+^ integrin binding to neutrophil ICAM-1 promotes its transition to E^+^H^+^. (**a**) Schematic showing the hypothesis that the *cis* interactions of E^−^H^+^ integrin (both LFA-1 and Mac-1) and neutrophil ICAM-1 may stabilize the E^−^H^+^ integrin. (**b**) Integrin clusters (E^+^H^−^ red, E^−^H^+^ green, E^+^H^+^ yellow) on arresting neutrophils rolling on P-selectin/ICAM-1/IL-8 with or without neutrophil ICAM-1 blocking by mAbs HA58 and R6.5; scale bar, 5 μm. (**c**–**e**) ICAM-1 blocking decreased the number of E^−^H^+^ clusters at arrest (**c**, *n*=15 cells). The number of E^+^H^+^ (**d**, *n*=15 cells) and E^+^H^−^ clusters (**e**, *n*=15 cells) at arrest with or without ICAM-1 blocking. (**f**) Dynamics of E^−^H^+^ clusters with or without ICAM-1 blockade on cells rolling on P-selectin/ICAM-1/IL-8. (**g**,**h**) ICAM-1 blocking decreased the duration of E^−^H^+^ clusters before transitioning to E^+^H^+^ clusters (**g**, *n*=16 clusters). Duration histograms (**h**, bin=1 s). Mean values and s.e.ms (error bars) were presented in (**c**–**e**) and (**g**). Log Gaussian (ICAM-1 blk) and Lorentzian (isotype) fits were used in **h**. n.s. *P*>0.05, ** *P*<0.01, *** *P*<0.001, **** *P*<0.0001 in unpaired student *t*-test. n.s., not significant.

Having shown that E^−^H^+^ neutrophil β_2_ integrins directly bind ICAM-1 in *cis*, we reasoned that this binding may stabilize E^−^H^+^ clusters. Thus, E^−^H^+^ clusters should be decreased when ICAM-1 binding to LFA-1 (using mAb HA58) and Mac-1 (using mAb R6.5) were blocked (Fig. 6a). Indeed, blocking ICAM-1 binding in *cis* (ICAM-1 blk) reduced the number of E^−^H^+^ clusters (Fig. 6b,c) at the time of neutrophil arrest (0 s). We found no significant difference in E^+^H^+^ (Fig. 6d) or E^+^H^−^ (Fig. 6e) clusters when ICAM-1 was blocked on the neutrophils, but ICAM-1 blk neutrophils reach the number of E^+^H^+^ clusters needed for arrest faster. Under control conditions, the number of E^−^H^+^ clusters increased with time, and this did not happen when ICAM-1 was blocked (Fig. 6f). If indeed β_2_ integrin interaction with ICAM-1 in *cis* stabilized the E^−^H^+^ conformation, then the duration of E^−^H^+^ clusters (time before having E^+^H^+^ on the cluster) should be reduced. Indeed, the average duration of E^−^H^+^ clusters was reduced from >5 s to <2 s (Fig. 6g,h).

### E^−^H^+^ β_2_ integrins inhibit neutrophil adhesion

**Figure 7:**
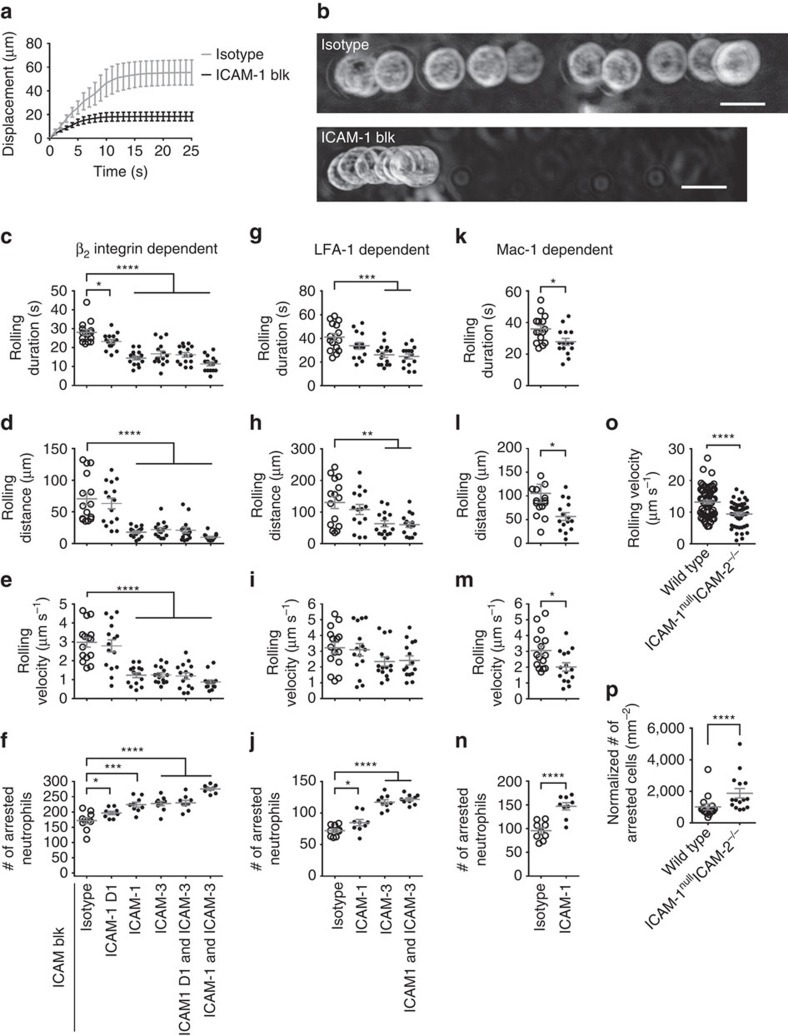
Blocking E^−^H^+^ integrin binding to neutrophil ICAMs promotes leukocyte adhesion. (**a**) Displacements of human neutrophils (*n*=5) with or without blockade of neutrophil ICAM-1 during rolling on P-selectin/ICAM-1/IL-8. (**b**) Maximum intensity projection of a typical bright-field-imaged human neutrophil with (13 frames) or without (30 frames) blockade of neutrophil ICAM-1 rolling on P-selectin/ICAM-1/IL-8. Flow direction is from left to right. Scale bar, 10 μm. (**c**–**f**) Rolling duration until arrest (**c**, *n*=15 cells per group), rolling distance until arrest (**d**, *n*=15 cells per group), rolling velocity before arrest (**e**, *n*=15 cells per group) and the number of arrested human neutrophils (**f**, *n*=9 observations per group) with or without blockade of neutrophil ICAM-1 D1 (domain 1, binds LFA-1, mAb HA58), or ICAM-1 (binds both LFA-1 and Mac-1, mAbs HA58 and R6.5), and/or ICAM-3 (binds LFA-1, mAb CBR-IC3/1). (**g**–**j**) Upon blockade of Mac-1 by mAb ICRF44, LFA-1-dependent *cis* binding was analysed with or without blockade of LFA-1 *cis* binding to neutrophil ICAM-1 (mAb HA58), and/or ICAM-3 (mAb CBR-IC3/1). *n*=15, 15, 15 and 9 cells per group in **g**,**h**,**i** and **j**, respectively. (**k**–**n**) Upon blockade of LFA-1 (mAb TS1/22), Mac-1-dependent *cis* binding was analysed with or without blockade of Mac-1 *cis* binding to neutrophil ICAM-1 (mAb R6.5). *n*=15, 15, 15 cells, and nine observations per group in **k**,**l**,**m** and **n**. (**o**) Leukocyte rolling velocity (*n*=48 for ICAM-1^null^ICAM-2^−/−^ and 65 for wild type^DsRed^ cells) in mouse cremaster muscle venules of ICAM-1^null^ICAM-2^−/−^/wild type^DsRed^ mixed chimeric mice before intravenous injection of CXCL1. (**p**) The number of adherent leukocytes (*n*=15 observations) in mouse cremaster muscle venules of the mixed chimeric mice 1 min after intravenous injection of 600 ng CXCL1. Mean values and s.e.ms (error bars) were presented in **a**,**c**–**p**. *P<0.05, **P<0.01, *** *P*<0.001, **** *P*<0.0001 in unpaired student *t*-test for **a**,**c**–**o** and paired student *t*-test for **p**.

Since β_2_ integrin interaction with ICAM-1 in *cis* stabilized the E^−^H^+^ conformation, we hypothesized that this may represent an auto-inhibitory pathway, because E^−^H^+^ integrins are not available for ligand binding in trans and thus are not expected to support cell adhesion under flow. Indeed, when ICAM-1 was blocked, human neutrophils arrested much quicker and rolled for a shorter distance than untreated human neutrophils on P-selectin/ICAM-1/IL-8 substrate (Fig. 7a,b). Human neutrophils express ICAM-1, a ligand for LFA-1 and Mac-1, and ICAM-3, a ligand for LFA-1, but not ICAM-2 (Supplementary Fig. 11). Therefore, we tested the rolling duration and distance (until arrest, Fig. 7c,d, respectively), the rolling velocity (Fig. 7e) and the resulting number of arrested neutrophils (Fig. 7f) with or without ICAM-1 and/or ICAM-3 blockade. Blocking ICAM-1 domain 1 (the LFA-1-binding site) was sufficient to make neutrophils adhere significantly faster (shorter rolling duration), resulting in more arrested neutrophils. Blocking both LFA-1 and Mac-1-binding sites on ICAM-1 further accelerated neutrophil accumulation. The effect of blocking ICAM-1 was similar to the effect of blocking ICAM-3, and blocking both ICAM-1 and ICAM-3 further reduced rolling duration, distance and velocity and further increased the number of arrested neutrophils.

To specifically look at the contributions of LFA-1 and Mac-1, we blocked Mac-1 by mAb ICRF44 (Fig. 7g–j) or LFA-1 by mAb TS1/22 (Fig. 7k–n). Since LFA-1 binds both ICAM-1 and ICAM-3, we tested the contribution of both ligands and found that blocking ICAM-3 alone had a stronger effect on the number of arrested neutrophils than blocking ICAM-1 alone. This is consistent with the higher expression of ICAM-3 than ICAM-1 on human neutrophils (Supplementary Fig. 11). We conclude that ICAM-3 is the dominant LFA-1 ligand in *cis*. Since Mac-1 binds only to ICAM-1, we tested its contribution by blocking the Mac-1 binding to ICAM-1 using mAb R6.5 (ref. 49). This intervention significantly decreased rolling duration, distance and velocity and significantly increased Mac-1-dependent neutrophil arrest. These data show that Mac-1 interaction with ICAM-1 in *cis* significantly contributes to limiting neutrophil accumulation.

To directly address the *in vivo* relevance of our findings, we performed *in vivo* mouse experiments. ICAM-1 and 2 are both expressed on mouse neutrophils^16^,^50^. We performed *in vivo* cremaster imaging on mice reconstituted with mixed ICAM-1/2-double-knockout^51^,^52^ and DsRed-wild type (1:1) bone marrow. ICAM-1/2-double-knockout leukocytes (non-fluorescent) showed significantly decreased rolling velocity in cremaster venules (Fig. 7o). After injecting the chemokine CXCL1, the number of arrested ICAM-1/2-double-knockout leukocytes was twice that of wild-type neutrophils (Fig. 7p). These data show that the binding of E^−^H^+^ β_2_ integrin to ICAM-1 and 2 in *cis* strongly inhibits leukocyte adhesion *in vivo*.

Based on the finding that integrin activation blockade by interaction of E^−^H^+^ β_2_ integrins with ICAMs in *cis* is relevant *in vitro* and *in vivo*, we asked whether it would also limit neutrophil aggregation. To test this, we performed an aggregation assay (Supplementary Fig. 12), where we stained human neutrophils with two different dyes (carboxyfluorescein succinimidyl ester (CFSE) and cell tracker orange (CMRA)) and tested the aggregation between the two populations. When ICAMs were blocked on the CMRA population, thus effectively blocking the *cis* interaction and liberating β_2_ integrins, the percentage of heteroaggregates increased about threefold. When we further blocked β_2_ integrins on the other (CFSE) population, which released the *cis*-binding ICAMs, CFSE-CMRA aggregates increased by a further factor of two. Therefore, without the inhibition of integrin extension by binding ICAMs in *cis*, neutrophil aggregation would be expected to be sixfold higher than it actually is. These results directly demonstrate that the *cis* interaction between E^−^H^+^ β2 integrin and ICAMs provides a relevant mechanism that inhibits neutrophil aggregation in suspension.

Taken together, our data support a new model (Supplementary Fig. 1b) where resting E^−^H^−^ LFA-1 and Mac-1 are stimulated by chemokine to assume the E^−^H^+^ conformation that binds mAb24, but not KIM127. This conformation is stabilized by interaction with ICAM-1 on the neutrophil in *cis*. When extension occurs, this converts E^−^H^+^ to E^+^H^+^ integrin, which is now able to bind ICAM-1 in trans (on the substrate, endothelium or other leukocytes) and thus promote arrest.

## Discussion

Here we elucidate the molecular mechanism of β_2_ integrin-dependent neutrophil arrest. As expected, rolling neutrophils express some β_2_ integrins in the E^+^H^−^ conformation. Unexpectedly, the E^+^H^−^ integrins are organized in clusters with an average size of ∼25 pixels (∼0.1 μm^2^). Unlike bulk β_2_ integrins, most of these E^+^H^−^ clusters are on the tips of microvilli and thus able to reach ICAM-1 on the flat substrate. *In vivo*, neutrophil hills and valleys may interdigitate with endothelial hills and valleys, but this cannot be imaged with sufficient resolution by intravital microscopy. Very few clusters of high-affinity (E^+^H^+^) integrin are observed on rolling neutrophils. When immobilized chemokine is added, both E^−^H^+^ and E^+^H^+^ clusters are induced. When the number of E^+^H^+^ clusters reaches ∼9 (∼7 within 50 nm from substrate), the cell stops rolling and arrests. We estimate that 258 fully activated E^+^H^+^ β_2_ integrin molecules are sufficient to arrest a human neutrophil at 6 dyn cm^−2^.

According to the switchblade model^23^,^25^ of integrin activation, E^+^ precedes H^+^. Thus, the appearance of E^−^H^+^ clusters was completely unexpected. The E^−^H^+^ conformation was suggested by an electron microscopic image of α_V_β_3_ integrin^53^, a crystal structure of α_X_β_2_ stabilized by introduced disulfide bridges^54^, and three Mac-1 mutants^55^. However, these first two constructs lacked transmembrane and cytoplasmic domains and the third introduced physiologically irrelevant mutations. Thus, the physiological significance remained unclear. Here we show that the E^−^H^+^ integrin conformation exists on primary human cells. Our findings suggest that integrin H^+^ and E^+^ are regulated individually, which disagrees with the canonical switchblade model^25^, where integrin H^+^ requires integrin extension (E^+^). We show that PSGL-1 signalling is sufficient to induce E^+^, whereas chemokine signalling necessary for H^+^.

Beyond these results that are relevant to our understanding of basic integrin biology, we discovered that E^−^H^+^ integrins bind ICAMs in *cis*, which effectively inhibits cell adhesion as evidenced by prolonged rolling distance, prolonged rolling time and reduced number of adherent neutrophils. This is a new, powerful and unexpected endogenous anti-inflammatory mechanism that impacts inflammation and immunity. This new mechanism not only limits neutrophil adhesion under flow, but also curbs neutrophil aggregation, which is associated with acute lung injury^56^, sepsis^57^ and coronary artery disease^58^. E^−^H^+^ LFA-1 binds to both ICAM-1 and ICAM-3 in *cis*; E^−^H^+^ Mac-1 binds to ICAM-1 in *cis*. Thus, both major β_2_ integrins on neutrophils are inhibited by binding ICAMs in *cis*. The FRET assay provides direct evidence of close proximity between H^+^E^−^ β_2_ integrins and ICAMs. The functional data (adhesion and aggregation assays) are consistent with our interpretation. Direct evidence to demonstrate the existence of the *cis* interaction of the β_2_ integrins with ICAMs might ultimately be provided by co-crystallization.

It is known that Mac-1 on neutrophils is increased upon chemokine stimulation by degranulation^33^. However, the degranulation does not occur until ∼5 min after chemokine. In our investigation, we considered the conformational changes of β_2_ integrins only less than 1 min after exposure to IL-8. Thus, Mac-1 degranulation does not influence our findings. Whether freshly recruited Mac-1 can interact with ICAMs in *cis* is unknown.

An alternative possibility that would explain the observation of KIM127^−^ mAb24^+^ clusters is steric exclusion. If extended integrin molecules were clustered so tightly that KIM127 cannot find its binding site, extended high-affinity integrin would be falsely classified as bent. If this were the case, clustered extended high-affinity integrin would be available to bind ligand in trans and not available to bind ligand in *cis*. Therefore, the steric antibody exclusion hypothesis is not supported by our functional data (Figs 6 and 7 and Supplementary Fig. 12), which clearly show that liberating β_2_ integrins from interactions in *cis* increases binding to ligand in trans and consequently neutrophil adhesion and aggregation.

Our finding also suggests a possible new class of drugs that target β_2_ integrins. Currently, six integrin-targeting drugs are on the market, five of which are antibodies or small molecules that inhibit the ligand-binding site^59^. The present data suggest that a new class of allosteric inhibitors that stabilize E^−^H^+^ may be useful as anti-inflammatory drugs. Conversely, molecules that release E^−^H^+^ to E^+^H^+^ could be used to fight infections where more neutrophil recruitment might be needed.

Beyond neutrophils, β_2_ integrins are widely expressed and functional in other leukocyte subsets. LFA-1 on lymphocytes is involved in trafficking to lymph nodes^60^ and in the formation of the immunological synapse^61^. The newly discovered E^−^H^+^ conformation thus may also be involved in the regulation of adaptive immunity. The recruitment of patrolling monocytes^62^ is also β_2_ integrin-dependent. Finally, other integrins may also have E^−^H^+^ conformations, which may be involved in other immunological processes, like β_1_ integrin-dependent^60^ or β_7_ integrin-dependent^63^ leukocyte recruitment.

In conclusion, we show that H^+^E^−^ β_2_ integrins exist on rolling neutrophils, where they bind ICAM-1 in *cis*, thus limiting neutrophil adhesion by preventing ICAM-1 binding in trans. These data support a revised model of β_2_ integrin activation separating headpiece opening from extension (Supplementary Fig. 1). Beside the significance in β_2_ integrin activation during neutrophil adhesion in particular, our finding provides insights into integrin function in general.

## Methods

### Reagents

Recombinant human P-selectin-Fc, ICAM-1-Fc and IL-8 were purchased from R&D Systems. Murine CXCL1 was purchased from Pepro Tech. Casein blocking buffer was purchased from Thermo Fisher Scientific. The conformation specific antibody mAb24 to human β_2_-I-like-domain, which reports the headpiece-opening^29^,^30^,^31^,^32^, was purchased from Abcam. The KIM127 mAb to human β_2_-IEGF-domain, which reports the ectodomain extension^27^,^28^, was purified at the Lymphocyte Culture Center at the University of Virginia from hybridoma supernatant (American Type Culture Collection). Purified CD11a blocking mAb TS1/22 was purchased from Thermo Fisher Scientific. Purified CD11b blocking mAb ICRF44, purified CD11a non-blocking mAb TS2/4, purified and FITC-conjugated ICAM-1 domain 1 mAb HA58, purified ICAM-3 blocking mAb CBR-IC3/1, purified ICAM-2 blocking mAb CBR-IC2/2, FITC-conjugated anti-mouse IgG_1_ mAb, and purified isotype control mAbs were purchased from Biolegend. The CD18 blocking mAb IB4, and human Fc receptor (FcR) blocking reagents were purchased from Miltenyi Biotec. Purified ICAM-1 domain 2 mAb R6.5 was purchased from eBioscience. FITC-conjugated CD14 mAb was purchased from Invitrogen. DL488- or DL550-conjugated isotype control mAbs were purchased from Novus Biologicals. FITC-conjugated isotype control mAbs was purchased from BD Bioscience. mAb24, KIM127 or TS2/4 were directly labelled by DL488 or DL550 using DyLight antibody labelling kits from Thermo Fisher Scientific. CellMask DeepRed was purchased from Molecular Probes. CellTrace CFSE and CellTracker Orange CMRA were purchased from Thermo Fisher Scientific. Quantum Simply Cellular anti-Mouse (calibration beads) was purchased from Bangs Laboratories. Polymorphprep was purchased from Accurate Chemical. Roswell Park Memorial Institute (RPMI) medium 1,640 without phenol red and phosphate-buffered saline (PBS) without Ca^2+^ and Mg^2+^ were purchased from Gibco. Human serum albumin (HSA) was purchased from Gemini Bio Products. Paraformaldehyde (PFA) was purchased from Thermo Fisher Scientific.

### Mice

C57BL/6J wild-type mice (000664; JAX) and DsRed mice (006051; JAX; wild-type C57BL/6J background) were obtained from the Jackson Laboratory. Mice were fed a standard rodent chow diet and were housed in microisolator cages in a pathogen-free facility. Mice were euthanized by CO_2_ inhalation. All experiments followed guidelines of the La Jolla Institute for Allergy and Immunology Animal Care and Use Committee, and approval for use of rodents was obtained from the La Jolla Institute for Allergy and Immunology according to criteria outlined in the Guide for the Care and Use of Laboratory Animals from the National Institutes of Health.

ICAM-1^null^ and ICAM-2^−/−^ mice were described before^51^,^52^. They were backcrossed to the C57BL/6 background for at least eight generations^64^. ICAM-1 and ICAM-2 double-deficient (ICAM-1^null^/ ICAM-2^−/−^) C57BL/6 mice were generated by crossing ICAM-1^null^ and ICAM-2^−/−^ C57BL/6 mice in the specific pathogen-free animal facility, University of Bern, Switzerland. Animal procedures were performed in accordance with the Swiss legislation on the protection of animals and approved by the veterinary office of the Kanton of Bern.

### Bone marrow transplantation for mixed chimera

Recipient mice (C57BL/6J wild-type male, 8-weeks old, Jackson Labs) were irradiated in two doses of 550 rads each (for a total of 1,100 rads) 4 h apart. Bone marrow cells from both femurs and tibias of donor mice (ICAM-1^null^ICAM-2^−/−^ male and DsRed wild-type male, 11 weeks old) were collected under sterile conditions. Bones were centrifuged for the collection of marrow, unfractionated bone marrow cells were washed, resuspended in PBS, mixed at a ratio of 1:1 (2.5 million cells each) in 200 μl, confirmed by flow cytometry and injected retro-orbitally into the lethally irradiated mice. Recipient mice were housed in a barrier facility under pathogen-free conditions before and after bone marrow transplantation. After bone marrow transplantation, mice were provided autoclaved acidified water with antibiotics (trimethoprim-sulfamethoxazole) and were fed autoclaved food. Mice were used for experiments 6 weeks after bone marrow reconstitution.

### Neutrophil isolation

Heparinized whole blood was obtained from healthy human donors after informed consent, as approved by the Institutional Review Board of the La Jolla Institute of Allergy & Immunology in accordance with the Declaration of Helsinki. Informed consent was obtained from all donors. Neutrophils were isolated by using Polymorphprep (a mixture of sodium metrizoate and Dextran 500) density gradient. Briefly, human blood was applied onto Polymorphprep, centrifuged at 500*g* for 35 min at 20–25 °C, resulting in neutrophils concentrated in a layer between peripheral blood mononuclear cells and erythrocytes. After washing with PBS without Ca^2+^ and Mg^2+^ twice, the neutrophils (>95% purity by flow cytometry, no visible activation by microscopy) were re-suspended in RPMI-1640 without phenol red plus 2% HSA and were used within 4 h. Neutrophils were incubated with FcR blocking reagents for 10 min at room temperature before all the experiments.

### Microfluidic device

The assembly of the microfluidic devices used in this study and the coating of coverslips with recombinant human P-selectin-Fc, ICAM-1-Fc and IL-8 has been described previously^15^,^16^,^33^. Briefly, coverslips were coated with P-selectin-Fc (2 μg ml^−1^), ICAM-1-Fc (10 μg ml^−1^), and IL-8 (10 μg ml^−1^) for 2 h and then blocked for 1 h with casein (1%) at room temperature. In some experiments (Fig. 2), coverslips were coated with P-selectin-Fc only, P-selectin-Fc plus ICAM-1-Fc, or P-selectin-Fc plus IL-8. After coating, coverslips were sealed to polydimethylsiloxane chips by magnetic clamps to create flow chamber channels ∼29 μm high and ∼300 μm across. By modulating the pressure between the inlet well and the outlet reservoir, 6 dyn cm^−2^ wall shear stress was applied in all experiments.

### Microfluidic perfusion assay

To study the arrest of neutrophils, isolated human primary neutrophils (5 × 10^6^ cells ml^−1^) were perfused in the microfluidic device over a substrate of recombinant human P-selectin-Fc with or without recombinant human ICAM-1-Fc and/or IL-8 under shear stress of 6 dyn cm^−2^. In some experiments (Supplementary Fig. 2a), neutrophils were incubated with anti-CD11a (TS1/22, blocking, 10 μg ml^−1^) mAb, anti-CD11b (ICRF44, blocking, 10 μg ml^−1^) mAb, anti-CD18 (IB4, blocking, 10 μg ml^−1^ mAb for 20 min at room temperature before being perfused into the microfluidic devices, as described previously^33^. In some experiments (Supplementary Fig. 2b–e), neutrophils were incubated with isotype mAb (10 μg ml^−1^), KIM127 and isotype (5 μg ml^−1^ each), mAb24 and isotype (5 μg ml^−1^ each) or KIM127 and mAb24 (5 μg ml^−1^ each) for 3 min at room temperature before being perfused into the microfluidic devices. In ICAM-1/ICAM-3 blocking experiments (Fig. 7c–n), neutrophils were incubated with ICAM-1 domain 1 blocking mAb HA58 (10 μg ml^−1^), or/and domain 3 blocking mAb R6.5 (10 μg ml^−1^), and/or ICAM-3 blocking mAb CBR-IC3/1 (10 μg ml^−1^), or/and isotype control mAbs for 20 min at room temperature, with two washes before being perfused into the microfluidic devices. IB4, TS1/22 and ICRF44 mAbs were used in some data sets to assess the contribution of β_2_ integrins, LFA-1 and Mac-1 in the *cis* interactions. The microfluidic devices were perfused with neutrophils for 10 min and washed with RPMI-1640 without phenol red plus 2% HSA for 5 min. Then, the arrested neutrophils were counted in nine fields-of-view per group. In some experiments, time-lapse images (one frame per second) were taken during the profusion. Then the rolling duration and rolling distance were acquired from the images by analysing 15 cells starting rolling to arrest.

### Homogeneous binding qDF imaging

The homogeneous binding assay (that is, the continuous real-time measurement without separation of soluble antibody^33^) and qDF imaging^15^,^16^ were combined here. Briefly, the conformation reporting antibody mAb24 or KIM127 were conjugated with DL488 or DL550, respectively, using the DyLight antibody labelling kits according to the manufacturer's instructions. In some experiments (Supplementary Fig. 7), the fluorochrmes of mAb24 and KIM127 were switched to test for possible nonspecific effects of the fluorochromes. In neutrophil ICAM-1 blocking experiments (Fig. 6), neutrophils were incubated with both ICAM-1 domain 1 mAb HA58 (10 μg ml^−1^) and domain 2 mAb R6.5 (10 μg ml^−1^), which will block both LFA-1 and Mac-1 binding^49^, or isotype control mAbs for 20 min at room temperature, with two washes before performing the homogeneous binding assay.

During the homogeneous binding assay, neutrophils (2.5 × 10^6^ cells ml^−1^) were incubated with fluorochrome-conjugated reporting mAbs (5 μg ml^−1^ each) for 3 min at room temperature and immediately perfused through the microfluidic device at a wall shear stress of 6 dyn cm^−2^ without separation of the soluble mAbs. The plasma membrane of neutrophils was labelled with CellMask DeepRed according to the manufacturer's instructions before the incubation with mAbs. When neutrophils were observed rolling on the substrate, acquisition was started using TqDF microscopy to acquire the dynamics of integrin activation on neutrophil footprint during rolling (∼30 s), arrest and ∼30–100 s following arrest.

To assess the binding kinetics and stability of binding of the mAbs, neutrophils were perfused through the microfluidic device at a wall shear stress of 6 dyn cm^−2^ for 5 min to allow them to roll and arrest on the substrate. Then 1% PFA was perfused through the device to fix the neutrophils for 5 min. After washing the device by PBS perfusion for 5 min, 5 μg ml^−1^ of fluorochrome-conjugated reporting mAbs KIM127 or mAb 24 were perfused through the device. Footprints of the fixed cells were recorded by TqDF microscopy.

### TqDF microscopy

The qDF set up and the theory of qDF have been described previously in detail^15^. Here, we expended qDF to three channels (TqDF). The set up consisted of an IX71 inverted TIRF research microscope (Olympus America) with a 100 × NA 1.45 plan-apochromatic oil immersion TIRF microscopy objective and 10 mW blue (*λ*=488 nm), 10 mW yellow-green (*λ*=561 nm), and 5 mW red (*λ*=641 nm) diode-pumped solid-state lasers (CVI Melles Griot) as TIRF excitation light sources. Images were captured at a rate of 0.2–1 frames per second using a QV2 (Photometrics) QuadView video coupler and a 16-bit digital charge coupled device camera (Hamamatsu C10600-10B ORCA-R2). The laser shutters and camera were controlled with the SlideBook5.5 software (Intelligent Imaging Innovations). The absorption and emission peaks of the fluorochromes used in this study were, respectively, 493 and 518 nm for DL488, 562 and 576 nm for DL550, 649 and 666 nm for CellMask DeepRed. There was no bleed-through between channels. A TIRF incidence angle of *θ*=70° was used for all three lasers in all TqDF experiments.

### Intravital microscopy

For *in vivo* imaging of leukocyte rolling and arrest, intravital microscopy of postcapillary venules of the cremaster muscle was performed on five mixed chimeric mice as described before^36^,^41^. Anesthesia was induced with an intraperitoneal injection of ketamine hydrochloride (125 mg kg^−1^) and xylazine (12.5 mg kg^−1^), and was maintained with inhalation of isoflurane/oxygen gas mixture. The left carotid artery was cannulated with a polyethylene-10 tube (BD) and the left cremaster muscle was exteriorized and prepared for imaging through a × 20 water immersion objective with simultaneous bright-field and epifluorescent imaging, where the wild-type (DsRed) leukocytes appeared bright and the ICAM-1^null^ICAM-2^−/−^ leukocytes appeared dim. On each cremaster muscle, three different postcapillary venular sites with rolling leukocytes were recorded. After one minute of recording rolling, 600 ng murine CXCL1 was injected through the carotid cannula. The numbers of arrested DsRed wild-type and ICAM-1^null^ICAM-2^−/−^ leukocytes were counted within 2 min after injections. The arrest counts were normalized by the number of rolling cells before murine-CXCL1 injection and the square of imaged venules.

### Δ map and footprint binary images

The distance (Δ) from any region in the neutrophil footprint within ∼200 nm to the total internal reflective interface can be calculated from fluorescence intensity of membrane dye using the equation described previously^15^. Membrane fluorescence images (Supplementary Fig. 4a) were converted to Δ maps (Supplementary Fig. 4b) that encode Δ as pixel intensity, using the ‘Math' function in FIJI-ImageJ2. The neutrophil footprint binary images were generated from Δ maps by setting a threshold of 95 (the distance to the interface ⩽95 nm, Supplementary Fig. 4c), which excluded the background not associated with the footprint. The footprint outline images (Fig. 1b,c,f, Supplementary Fig. 4d,g and Supplementary Movie 1) were generated from footprint binary images using the ‘Outline' function in FIJI-ImageJ2.

### Displacements of the neutrophils and definition of the arrest

The time-lapse footprint binary images were used to compute the cell velocities and displacements (Fig. 2a–d) using ‘TrackMate' in FIJI-ImageJ2. Cell arrest was defined as the time when the velocity dropped below 0.1 μm s^−1^.

### Binary images of integrin clusters

Binary images of integrin clusters (Fig. 1d–f, Fig. 3a, Fig. 4d–f, Fig. 6b, Supplementary Figs 4f,g and 7 and Supplementary Movies 1 and 2) were generated from raw images (Supplementary Figs 4e,5) by using ‘Smart Segmentation' in ImagePro Premier 9.1 (Media Cybernetics), which is widely used for identifying objects^65^. Smart Segmentation is a pixel classification algorithm^66^, which uses reference objects to define classes based on pixel intensities. Subsequently, each pixel in the image is analysed and compared with the values of the reference objects and the pixel is assigned to the class of the closest reference object. Different from simple thresholding, Smart Segmentation can identify clusters by comparing each cluster with its local background. Final binary images for integrin clusters (Supplementary Fig. 4g) were prepared by subtracting background noise not associated with neutrophil footprints using ‘image calculator' in FIJI-ImageJ2.

Dual colour binary images of integrin clusters were split into binary images for yellow (E^+^H^+^), red (E^+^H^−^), and green (E^−^H^+^) clusters, respectively. Raw images were masked with the binary clusters and mean fluorescence intensity was quantified using the ‘analyze particles' function in FIJI-ImageJ2. The mean fluorescence intensities (MFI) were normalized by background intensities and highest fluorescence intensities in the recording. Quantification of the KIM127 and mAb24 MFI of yellow (E^+^H^+^, Supplementary Fig. 4h), red (E^+^H^−^, Supplementary Fig. 4i), or green (E^−^H^+^, Supplementary Fig. 4j) clusters demonstrated the accuracy of the cluster binary images generated by ‘Smart Segmentation'. KIM127 and mAb24 MFI for each cluster were plotted against each other to identify the three populations of clusters (Supplementary Fig. 4k). To measure the cluster number (Fig. 2e–t, Fig. 4m, Fig. 6c–f, Supplementary Fig. 6a), total area (Supplementary Fig. 6b) and average size (Supplementary Fig. 6c), the cluster binary images were analysed by ‘analyze particles' in FIJI-ImageJ2.

### Colour transition history of the clusters

The cluster binary images were analysed manually to reveal the colour transition history (representing integrin conformation changes) of the clusters. We analysed the E^+^H^+^ clusters after cell arrest. 15 clusters, which transitioned from E^+^H^−^ clusters (Fig. 3b,c), and 15 clusters, which transitioned from E^−^H^+^ clusters (Fig. 3d,e), were analysed by acquiring the pixel colours over 4 s. KIM127 and mAb24 fluorescence intensity of individual pixels in these clusters were analysed within these 4 s (Supplementary Fig. 8). In some analyses, 15 arrested cells were selected to reveal their colour transition history of the clusters (Fig. 3f). The colours when the clusters were first observed were defined as their initial colour. In some analyses, the durations of 16 E^−^H^+^ clusters each on ICAM-1 blocked or isotype mAb-treated neutrophils were calculated (Fig. 6g,h). The durations were the time from the appearing of the green clusters to appearing of yellow pixels in the clusters.

### Creation of 3D reconstructions/footprint topography

Raw CellMask DeepRed qDF images were used to create 3D reconstructions (3D topography, Fig. 4a–f, Supplementary Fig. 9 and Supplementary Movie 2) by custom scripts in Matlab (MathWorks) as described previously^15^. Hills (microvilli) and valleys (the space between microvilli) were identified from CellMask DeepRed images by using ‘Smart Segmentation' in ImagePro. Hills and valleys were psuedocoloured blue and magenta, respectively, to generate hill-valley maps (Fig. 4c, Supplementary Fig. 9) by custom scripts in Matlab.

### 3D localization of the clusters

To reveal the 3D localization of the clusters, the cluster binary images were superimposed on the 3D topography (Fig. 4d–f and Supplementary Movie 2) by custom scripts in Matlab. We masked the hill-valley maps and the Δ maps with the yellow (E^+^H^+^), red (E^+^H^−^), or green (E^−^H^+^) cluster areas, respectively, using ‘image calculator' in FIJI-ImageJ2. The hill or valley location of each cluster was analysed by using ‘measure' in FIJI-ImageJ2. Similarly, the Δ of every cluster Fig. 4j–l) was analysed by the ‘analyze particles' function in FIJI-ImageJ2.

### Integrin sites measurements

To measure the integrin expression/epitope sites, we used QuantumTM Simply Cellular anti-Rat IgG beads coated with anti-rat IgG antibodies at five known antibody binding capacities. In all, 10^6^ ml^−1^ human neutrophils were incubated with FcR blocking reagents for 10 min at room temperature, and then incubated with 5 μg ml^−1^ KIM127, mAb24, which are both mouse anti-human IgG_1_ isotype, and isotype control, respectively, for 5 min at room temperature. After fixation by 1% PFA on ice for 10 min, the cells were washed twice and incubated with FITC-conjugated rat anti-mouse IgG_1_ mAb on ice for 30 min. The calibration beads were incubated with the same FITC-conjugated rat anti-mouse IgG_1_ mAb according to the manufacturer's instruction. After measuring the fluorescence intensity of calibration beads, a linear correlation between the fluorescence intensity and antibody-binding sites was established.

KIM127 and mAb24 epitope sites on the footprint of rolling cells were determined by dividing the number of antibody molecules bound per cell by the cell surface and multiplying by the footprint area. Cell surface area was estimated based on the average diameter of 64 neutrophils captured by microscopy. The fluorescence intensity integral of both KIM127 and mAb24 on the footprint were calculated and converted to KIM127 and mAb24 epitope sites.

### FRET assay using flow cytometry

Flow cytometry with compensation (BD FACSDiva) for bleed-through based FRET were used for detecting molecular interaction with high sensitivity^35^,^67^. To test whether E^−^H^+^ integrin can interact with endogenous ICAM-1 in *cis*, FRET between H^+^ (mAb24-550 as acceptor) and ICAM-1 (domain 1 mAb HA58-FITC as donor) was measured. This assay tests the *cis* interaction of neutrophil ICAM-1 and E^−^H^+^ Mac-1, which binds ICAM-1 domain 3. Isolated neutrophils (10^6^ cells ml^−1^) were incubated with FcR blocking reagents (1:100) for 10 min at room temperature, followed by incubating with 5 μg ml^−1^ purified isotype control or mAb R6.5, which blocks Mac-1-ICAM-1-binding^49^, for 20 min at room temperature. Live cells were tested by time-resolved flow cytometry. The 488 nm laser excited the FRET donor HA58-FITC (525/50 nm), which excited the FRET acceptor mAb24-DL550 (575/25 nm).

To quantify the quenching of FRET donor fluorescence, HA58-FITC (2 μg ml^−1^) was added at 10 s after starting recording, with 3 min recording to reach saturation, followed by adding IL-8 (1 μg ml^−1^) inducing the mAb24 epitope (mAb24-DL550, 5 μg ml^−1^, Fig. 5b,d). mAb24-DL550 was replaced by vehicle, non-binding isotype control mAb (IgG_1_-DL550, 5 μg ml^−1^) or KIM127-DL550 (5 μg ml^−1^), respectively, as negative controls. ICAM-1 blocked neutrophils served as control to test whether the blockade of Mac-1-ICAM-1 in *cis* interaction will eliminate the quenching of FRET donor HA58-FITC.

To quantify the increase in fluorescence of the FRET acceptor, IL-8 and mAb24-DL550 (1.5 μg ml^−1^) were added at 10 s after starting recording, with 3 min recording to reach saturation, followed by adding HA58-FITC (2 μg ml^−1^, Fig. 5c,e). HA58-FITC was replaced by vehicle, isotype control mAb (IgG_1_-FITC, 2 μg ml^−1^) or anti-CD14-FITC (2 μg ml^−1^), respectively, as negative controls. ICAM-1 blocked neutrophils served as control to test whether the blockade of Mac-1-ICAM-1 in *cis* interaction will eliminate the fluorescence increase of FRET acceptor mAb24-DL550.

### Distance calculation of the FRET pairs

From the data obtained by the flow cytometry-based FRET assay, we obtain the value of FRET efficiency (*E*) as


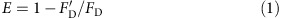


in which *F′*_D_ and *F*_D_ is the fluorescence intensity of the FRET donor (FITC) in the presence and in the absence of the FRET acceptor (DL-550), respectively.

*E* depends on the donor-to-acceptor separation distance r with an inverse sixth power law due to the dipole–dipole coupling mechanism:


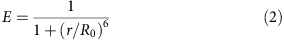


in which *R*_0_ is the Förster distance of this pair of donor and acceptor, defined as the distance at which the energy transfer efficiency is 50%. *R*_0_ depends on the overlap integral of the donor emission spectrum with the acceptor absorption spectrum and their mutual molecular orientation as expressed by the following equation:


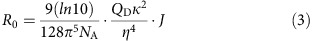


in which *N*_A_ is Avogadro's number; *Q*_D_ is the fluorescence quantum yield of the donor in the absence of acceptor, which is 0.92 of FITC in physiological solutions^68^; *κ*^2^ is the dipole orientation factor (2/3 when both dyes are freely rotating and isotropically oriented); *η* (refractive Index) of medium is ∼1.338 (ref. 69) and *J* is the spectral overlap integral of the donor-acceptor pair. The calculation of *J* was done by using a|e-ultraviolet-Vis-infrared Spectral Software 2.2 (FluorTools, www.fluortools.com). Spectra were obtained from Thermo Fisher Scientific.

### Aggregation assay using flow cytometry

To assess whether the *cis* binding of β_2_ integrins to ICAMs affects neutrophil aggregation, we performed a flow cytometry based aggregation assay^70^. We separated isolated neutrophils into two populations labelled with CFSE and CMRA, respectively. Aggregation of a CFSE-labelled neutrophil with a CMRA-labelled neutrophil appears as a double-positive event in flow cytometry. ICAM-1 (10 μg ml^−1^ HA58 and 10 μg ml^−1^ R6.5), ICAM-2 (10 μg ml^−1^ CBR-IC2/2) and ICAM-3 (10 μg ml^−1^ CBR-IC3/1) were all blocked (room temperature, 30 min) to eliminate the *cis* binding on CMRA population. In some experiments, β_2_ integrins on the CFSE population were blocked by 10 μg ml^−1^ IB4 at room temperature for 30 min.

### Statistics

Statistical analysis was performed with Prism 6 (GraphPad). Data (Fig. 2a–d,f–h,j–l,n–p,r–t, Fig. 3b–f, Fig. 4j–l, Fig. 5d,e, Fig. 6c–e,g, Fig. 7a,c–p, Supplementary Figs 2,3,4h–j and 6) are presented as mean±s.e.m. Single data points are presented in some graphs (Fig. 2e–t, Fig. 3f, Fig. 4j–m, Fig. 6c–h, Fig. 7c–p, Supplementary Fig. 2,4h–k and 6). The means for the data sets were compared using paired (Fig. 7p) or unpaired student *t*-tests with equal variances (*n*<30, Fig. 2f–h,j–l,n–p, r–t, Fig. 3f, Fig. 4j–l, Fig. 5d,e, Fig. 6c–e,g, Fig. 7c–n, Supplementary Fig. 2) or Chi-square test (*n*⩾30, Fig. 7o). Log-Gaussian, Gaussian and Lorentizian fits were applied, and the best fit for the data sets were shown in Fig. 6h. Linear regression fits were applied for the data sets in Fig. 2a–d, Fig. 4g–i, Fig. 6f. The slopes of the linear regression for the data sets in Fig. 4g–i were tested against zero and the slopes of the linear regression for the data sets in Fig. 6f were tested against each other using an *F*-test. *P* values <0.05 were considered significant. *Post hoc* analysis (SPSS 22.0 software, IBM) was performed to ensure that the sample size we chose had adequate power.

### Data availability

The data that support the findings of this study are available from the corresponding author upon request.

## 

## Additional information

**How to cite this article:** Fan, Z. *et al*. Neutrophil recruitment limited by high-affinity bent β_2_ integrin-binding ligand in *cis*. *Nat. Commun.* 7:12658 doi: 10.1038/ncomms12658 (2016).

## Supplementary Material

Supplementary Figures Supplementary Figures 1-12.

Supplementary Movie 1 Dynamics of β2 integrin extension (KIM127) and headpiece-opening (mAb24) during arrest of primary human neutrophils rolling on P-selectin, ICAM-1 and IL-8: Isolated primary human neutrophils were perfused through the microfluidic chamber (wall shear stress 6 dyn/cm^2^) coated with P-selectin, ICAM-1 and IL-8. A typical cell rolled for 30 s, then arrested at time = 0 s. Footprints of the cell (see Extended Data Fig. 3a-d) shown in grey outlines. The extended conformation of β_2_ integrins were identified by DL550 conjugated KIM127 (red), and the open headpiece conformation of β_2_ integrins were identified by DL488 conjugated mAb24 (green). Binary images; scale bar 5 μm. Clusters classified as E^+^H^-^ (red), E^-^H^+^ (green), and E^+^>H^+^ (yellow).

Supplementary Movie 2 3D distributions of different β2 integrin activation clusters in primary human neutrophils arresting on P-selectin, ICAM-1 and IL-8. Binary images of E^+^H^-^ (red), E^-^H^+^ (green) or E^+^H^+^ (yellow) clusters overlaid with 3D topography of neutrophil footprint, derived from membrane fluorescence signal as in Fig. 4a and presented as hills (microvilli) and valleys (space between microvilli). Rotations of 3D topography with clusters at the time of arrest (time = 0) about the flow axis (x).
