# Supplementary material for: Neutrophil recruitment limited by high-affinity bent β2 integrin binding ligand in cis
Source: Nat Commun. 2016 Aug 31;7:12658. doi: 10.1038/ncomms12658 (PMC5013657; doi:10.1038/ncomms12658)
Supplement: Supplementary Figures — 1-12. [file ncomms12658-s1.pdf]

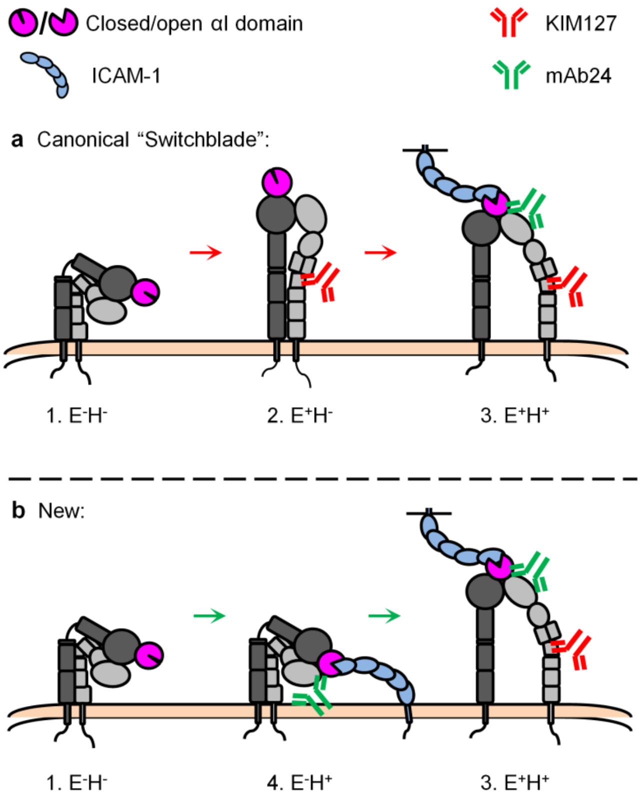

Supplementary Figure 1

Two activation pathways and four conformations of β_2 integrins. KIM127 (red) can specifically detect integrin extension (E^+) and mAb24 (green) can specifically detect headpiece-opening (H^+). (**a**) Canonical switchblade pathway: E^-H^- (**1**, $KIM127^-mAb24^-$) $\rightarrow E^+H^-$ (**2**, $KIM127^+mAb24^-$), $\rightarrow E^+H^+$ (**3**, $KIM127^+mAb24^+$); (**b**) Proposed new pathway: E^-H^- (**1**, $KIM127^-mAb24^-$) $\rightarrow E^-H^+$ (**4**, $mAb24^+KIM127^-$) $\rightarrow E^+H^+$ (**3**, $KIM127^+mAb24^+$).

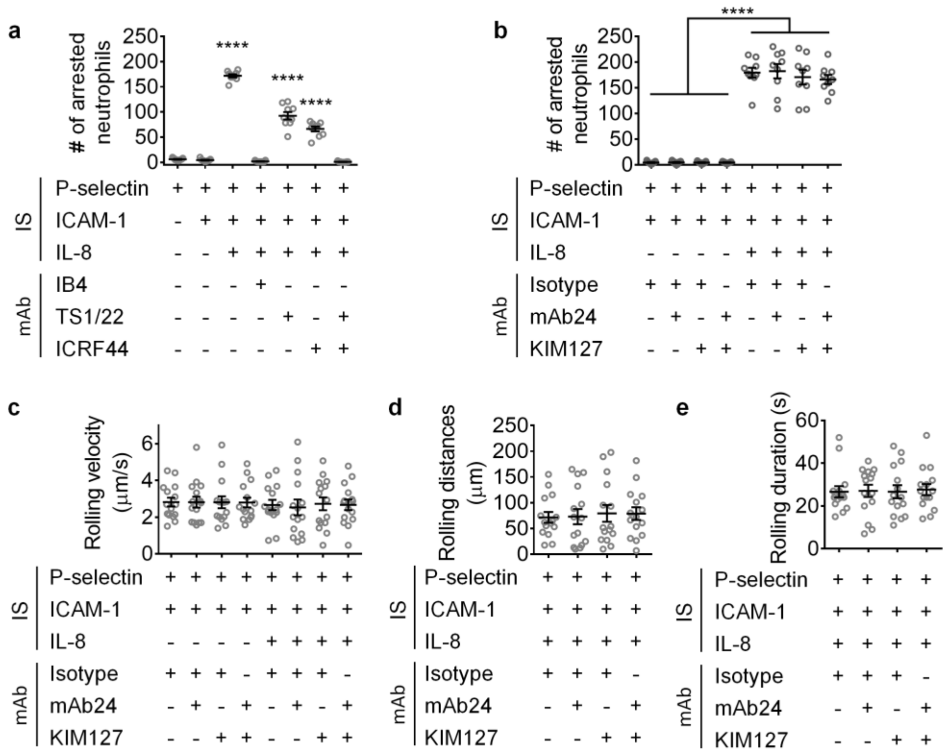

Supplementary Figure 2

Neutrophils roll on P-selectin and arrest when ICAM-1 and IL-8 are co-immobilized. Isolated primary human neutrophils ($5x10^6$ cells/ml) were perfused through the microfluidic device over a substrate coated with recombinant human P-selectin-Fc with or without recombinant human ICAM-1-Fc and IL-8 under shear stress of 6 dyn/cm^2. IS – immobilized substrate; mAb – soluble monoclonal antibodies. (**a**) Adhesion –blocking anti-CD11a (TS1/22), anti-CD11b (ICRF44), and anti-CD18 (IB4) mAbs (10 μg/ml each) were added to the cell suspension, incubated for 20 minutes at RT and then perfused with the cells as described previously[49]. (**b-e**) Neutrophils were incubated (3 min, RT, same as that used in homogeneous binding qDF imaging) with isotype control mAbs (10 μg/ml), mAb24/isotype (5μg/ml each), KIM127/isotype (5μg/ml each) and mAb24/KIM127 (5μg/ml each) prior to perfusion. n = 9 observations in **b**, n = 15 cells per group in **c-e**. The mean values (bars) and SEMs (error bars) are presented. ****$p<0.0001$ in the unpaired student t-test.

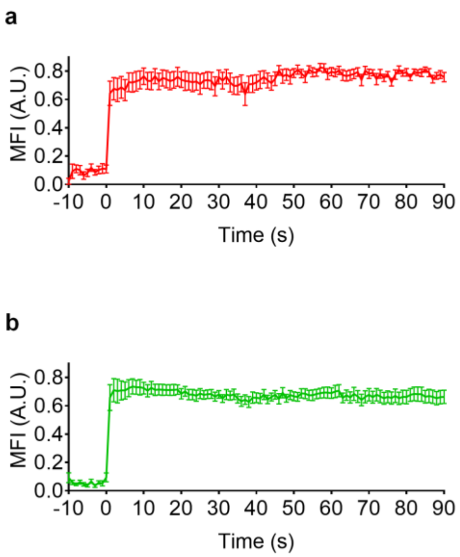

Supplementary Figure 3

Binding kinetics of KIM127-DL550 (**a**, red) and mAb24-DL488 (**b**, green) in qDF microscopy imaging. Unlabeled neutrophils (2.5×10^6 cells/ml) were perfused through the complete substrate (P-selectin/ICAM-1/IL-8) for 5 minutes to allow them arrest. Then the cells were fixed by PFA. After washing with PBS for 5 minutes, the KIM127-DL550 and mAb24-DL488 (5µg/ml each) antibodies were perfused to record the binding kinetics. MFI of both KIM127-DL550 and mAb24-DL488 on the cell footprints (n=16 cells) in the recorded time-lapse images were obtained. The binding of the antibodies is very fast as expected (reaching >90% of maximum binding within 1 second).

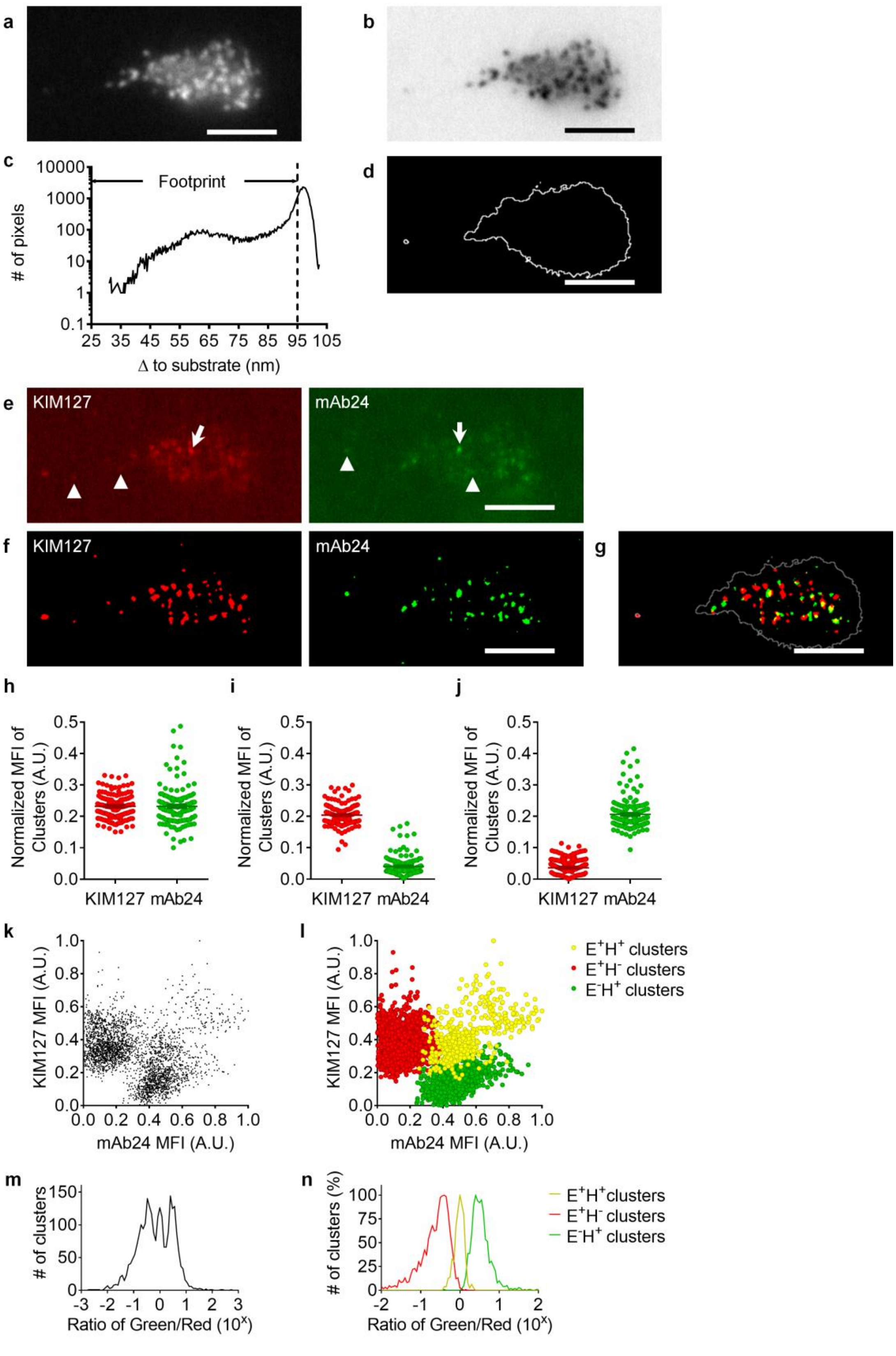
a
b
c
10000
1000
100
10
1
0.1
Footprint
# of pixels
25 35 45 55 65 75 85 95 105
Δ to substrate (nm)
d
e
KIM127
mAb24
f
KIM127
mAb24
g
h
i
j
Normalized MFI of Clusters (A.U.)
KIM127 mAb24
k
KIM127 MFI (A.U.)
mAb24 MFI (A.U.)
l
E+H+ clusters
E+H- clusters
E-H+ clusters
m
# of clusters
Ratio of Green/Red (10x)
n
# of clusters (%)
E+H+clusters
E+H- clusters
E-H+ clusters
Ratio of Green/Red (10x)

Supplementary Figure 4

Image processing: generation of neutrophil footprint outline and binary cluster images. (**a**) Raw fluorescence image of cell membrane labeled with CellMask DeepRed. (**b**) Distance between the membrane and the substrate (*Δ*) calculated from the fluorescence intensity of cell membrane dye as described previously[33] to get the *Δ* map. (**c**) Pixel histogram. The dashed line indentifies the footprint as being closer than 95 nm from the substrate. (**d**) Outline of the neutrophil footprint. (**e**) Raw image of KIM127-DL550 and mAb24-DL488. (**f**) Using "Smart Segmentation" in ImagePro (see methods), we generated binary cluster images, which identify both bright (arrows in **e**) and dim (arrow-heads in **e**) clusters in raw images. (**g**) The final binary cluster images only show the integrin clusters on cell footprints (grey outline). Scale bars in A, B and D-G are 5 μm. (**h-j**) MFI of KIM127-DL550 (red) and mAb24-DL488 (green) in E^+H^+ (**h**), E^+H^- (**i**) and E^-H^+ (**j**) clusters. In each frame, clusters were classified and their DL550 and DL488 fluorescence intensities were averaged, resulting in three data points (**h**, **j**, **k**) per frame. The mean values (bars) and SEMs (error bars) are presented. MFI = (intensity - background) / (maximum - background). (**k-l**) 2D plot KIM127 MFI (y-axis) vs mAb24 MFI (x-axis) of the 2506 clusters analyzed. Uncolored (**k**) and colored (**l**) plot showed that E^+H^- (red), E^-H^+ (green) and E^+H^+ (yellow) clusters clearly separated. (**m-n**) Histogram showing the ratio of mAb24 MFI vs KIM127 MFI of the 2506 clusters analyzed. Uncolored (**m**) and colored (**n**) histograms showed individual peaks for the E^+H^- (red), E^-H^+ (green) and E^+H^+ (yellow) clusters.

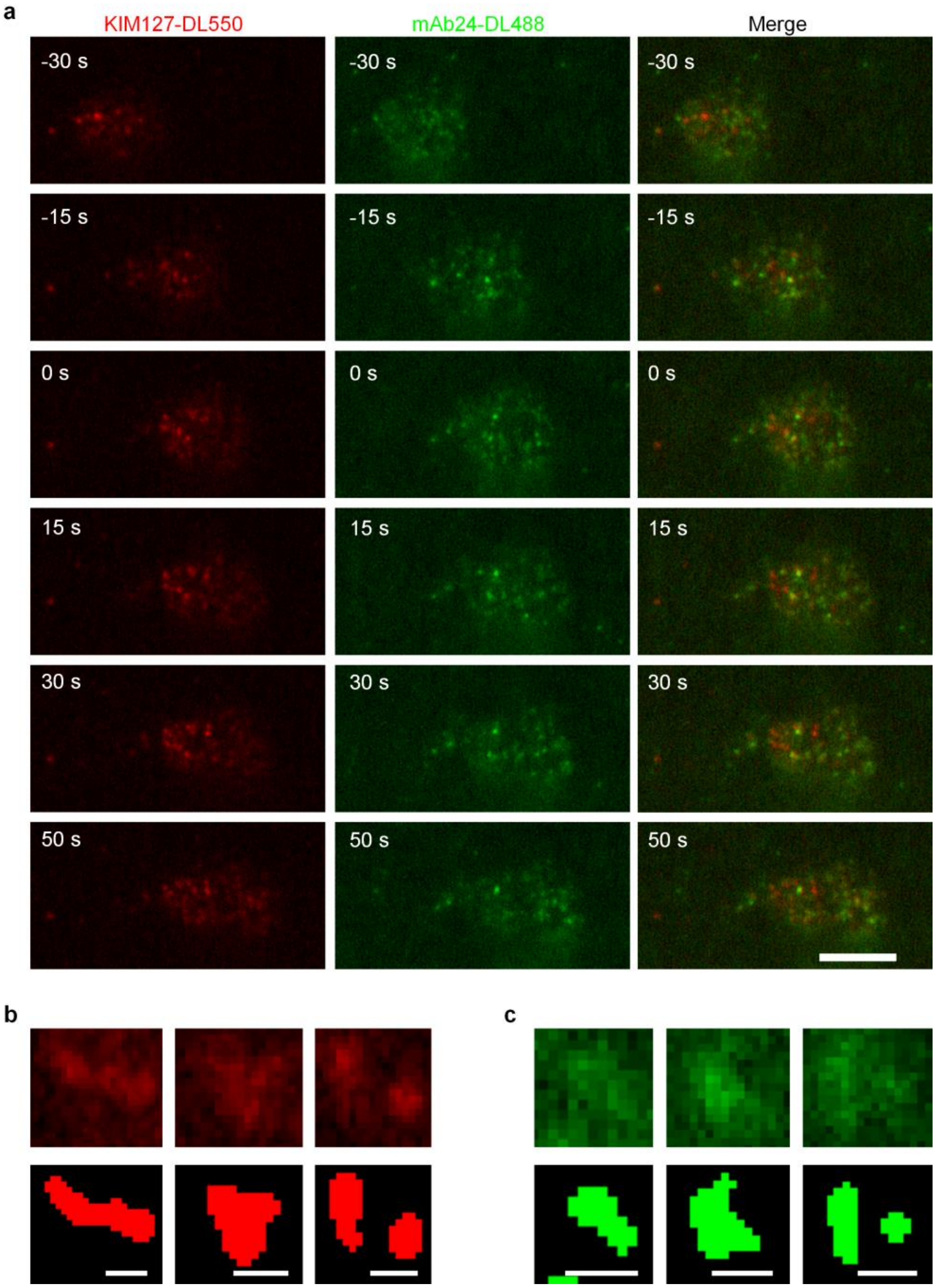
a
KIM127-DL550
mAb24-DL488
Merge
-30 s
-15 s
0 s
15 s
30 s
50 s
b
c

Supplementary Figure 5

Raw fluorescence images showing the β_2 integrin extension (KIM127) and headpiece-opening (mAb24) on human neutrophil footprint during rolling on P-selectin/ICAM-1/IL-8 substrate. (**a**) Raw fluorescence images used to generate Figure 1d-f. Scale bar 5 μm. (**b-c**) Comparison of raw fluorescence images and processed binary images for typical KIM127 (**b**) or mAb24 (**c**) clusters. Cluster morphologies are preserved in binary clusters. Scale bar 0.5 μm.

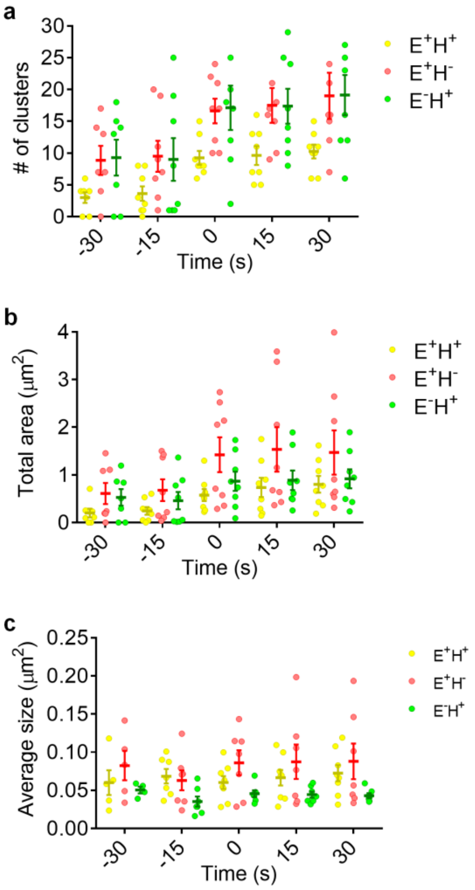

Supplementary Figure 6

Integrin clusters during neutrophil rolling and arrest on P-selectin, ICAM-1 and IL-8. Number (**a**), total area (**b**) and average size (**c**) of E^+H^+ (yellow), E^+H^- (red), and E^-H^+ (green) clusters over 15 seconds (n=8 cells). The mean values (bars) and SEMs (error bars) are presented. Each cell is represented by one dot. Arrest at time = 0 s.

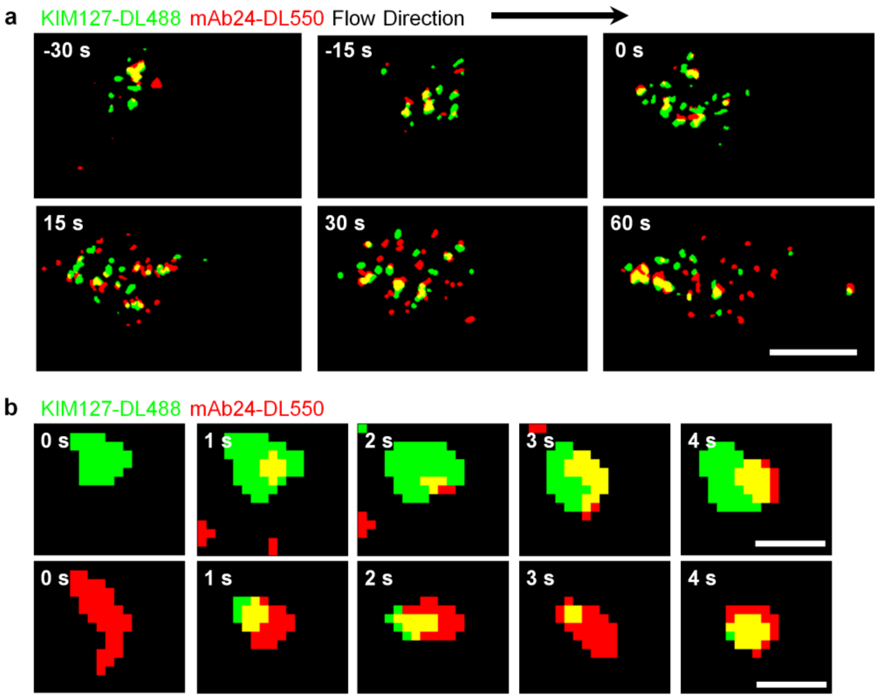

Supplementary Figure 7

Switching mAb-conjugations. (**a**) The extended conformation of β_2 integrins was identified by DL488 conjugated KIM127 (green), and the open headpiece conformation of β_2 integrins was identified by DL550 conjugated mAb24 (red). Binary images; Clusters can be identified as E^+H^+ (yellow), E^+H^- (green) or E^-H^+ (red). The clustering of the β_2 integrins and the increase in cluster number for all three antibody combinations were observed, similar to Fig. 1d; scale bar 5 μm. (**b**) The two pathways of β_2 integrin activation were still observed after switching mAb-conjugations: E^+H^- (green) or E^-H^+ (red) clusters both transitioned to E^+H^+ (yellow) clusters in 4 seconds as shown in Fig. 2a; scale bars 0.5 μm.

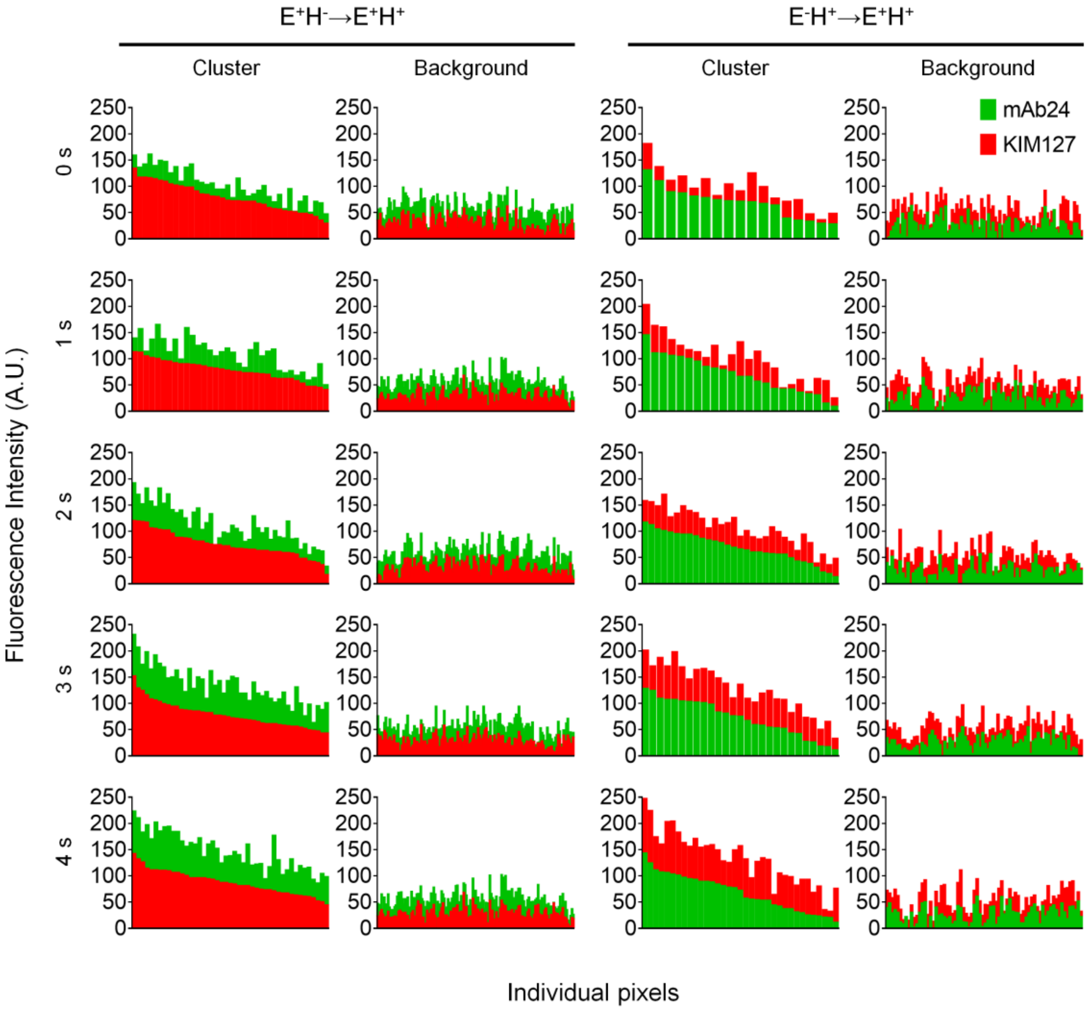

E+H-→E+H+
E-H+→E+H+
Cluster
Background
Cluster
Background
mAb24
KIM127
0 s
1 s
2 s
3 s
4 s
250
200
150
100
50
0
Fluorescence Intensity (A.U.)
Individual pixels

Supplementary Figure 8

Pixel statistics showing the transitions from one E^+H^- (left two columns) cluster and one E^-H^+ (right two columns) to E^+H^+ clusters within four seconds. Fluorescence intensities of both KIM127-DL550 and mAb24-DL488 in each individual pixels of clusters or non-cluster background were obtained. The background intensities in both transitions did not vary significantly over time. In the transition from E^+H^- to E^+H^+ cluster, KIM127-DL550 intensity remained similar,, whereas mAb24-DL488 intensity increased. In the transition from E^-H^+ to E^+H^+ cluster, mAb24-DL488 intensity remained similar, whereas KIM127-DL550 intensity increased. Each bar is one pixel.

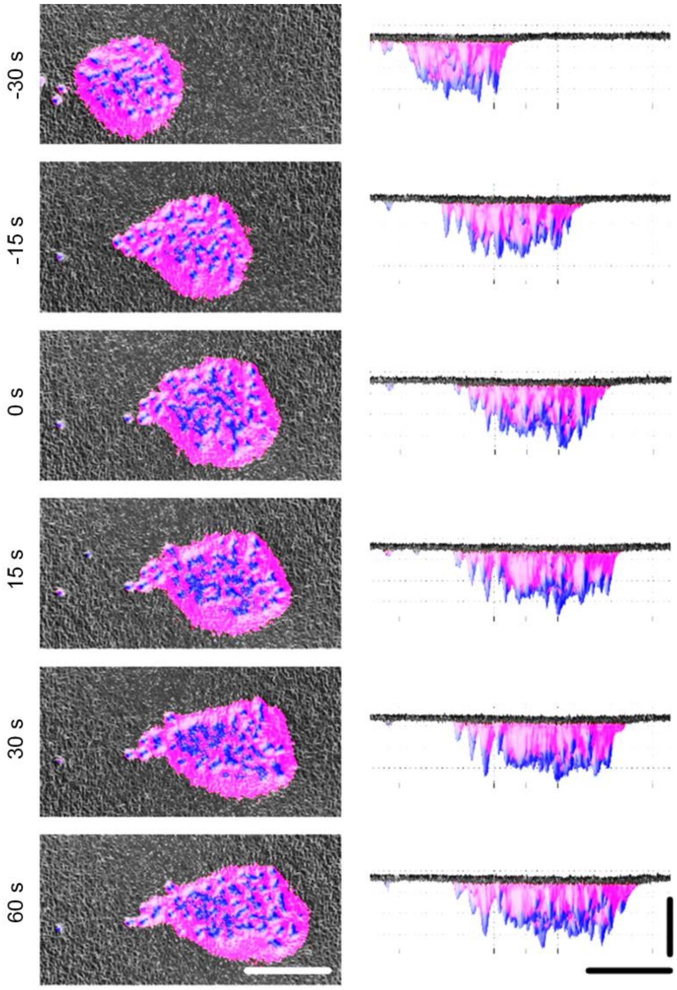

Supplementary Figure 9

Hills (blue) and valleys (magenta) identified on time-lapse 3D topography of neutrophil footprints during rolling (-30 to 0 second) and arrest (0 to 60 seconds). The hills and valleys were identified using "Smart Segmentation" in ImagePro as described in the experimental procedures section. Top-views (Left row), side-views (right row). Horizontal scale bars 5 μm, vertical scale bar 50 nm.

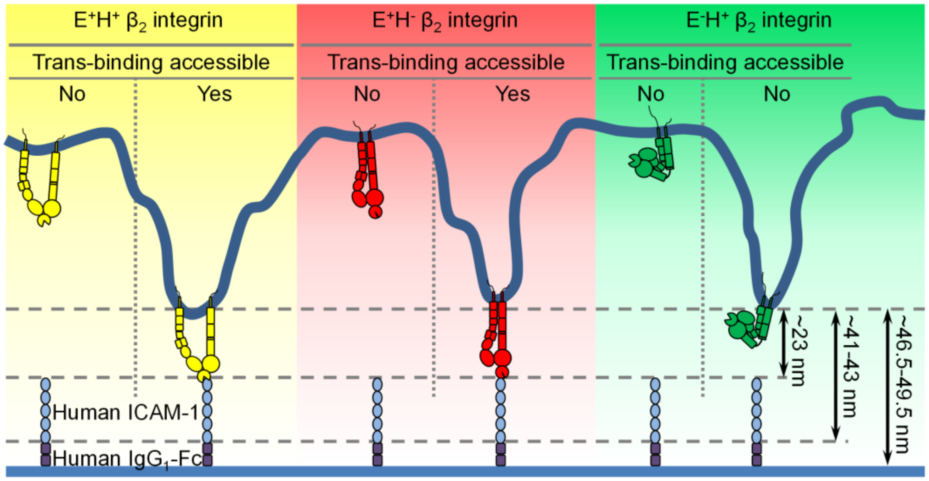

Supplementary Figure 10

Schematic showing the localization of the E^+H^+ (yellow), E^+H^- (red), or E^-H^+ (green) integrins accessibility for binding in trans.

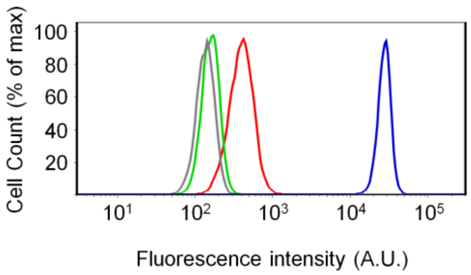

Supplementary Figure 11

ICAM-1, 2, and 3 expression on human neutrophils assessed by flow cytometry. Parallel samples of human neutrophils (10^6cells/ml) were incubated with isotype control (10 μg/ml), ICAM-1 mAb (HA58, 10 μg/ml), ICAM-2 mAb (CBR-IC2/2, 10 μg/ml) and ICAM-3 mAb (CBR-IC3/3, 10 μg/ml), respectively, at room temperature for 30 minutes. After staining with FITC-conjugated secondary antibody, the expression of ICAM-1, ICAM-2 and ICAM-3 was assessed. ICAM-1 (red) and ICAM-3 (blue) expressed, ICAM-2 (green) near isotype control (gray).

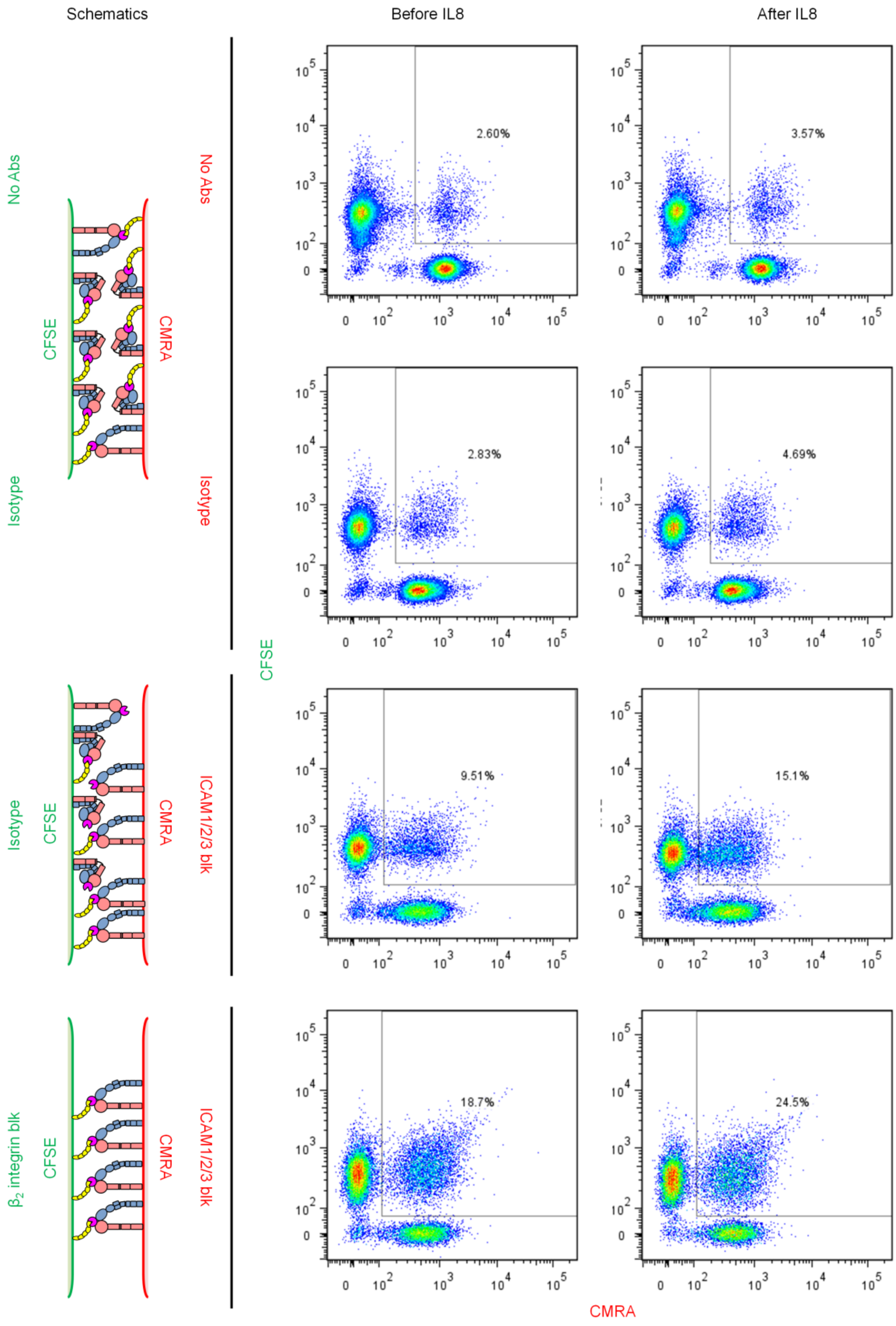
Schematics
Before IL8
After IL8
No Abs
Isotype
CFSE
CMRA
No Abs
Isotype
ICAM1/2/3 blk
β_2 integrin blk
2.60%
3.57%
2.83%
4.69%
9.51%
15.1%
18.7%
24.5%
CFSE
CMRA

Supplementary Figure 12

Blocking the cis interactions of E^-H^+ integrin with neutrophil ICAMs promotes neutrophil aggregation. Neutrophil suspension from one donor was split in half and labeled with CFSE and CMRA, respectively. Top two rows: when the cis interactions of E^-H^+ integrin with ICAMs were not blocked (no Abs and Isotype controls), aggregation between the CFSE and CMRA labeled neutrophils is rare (~2-3% without IL-8, ~4-5% with IL-8). Row three: when the cis interactions of E^-H^+ integrin with ICAMs were blocked in one population (CMRA, HA58 and R6.5 for ICAM-1, CBR-IC2/2 for ICAM-2, CBR-IC3/1 for ICAM-3, 10μg/ml each), the aggregation between CFSE and CMRA labeled neutrophils increased (>3fold), to ~9.5% without IL-8 stimulation, and ~15% with IL-8, indicating that more trans bounds are formed when the cis interaction is eliminated. Bottom Row: Further blockade of β_2 integrins on the other (CFSE) population releases more ICAMs for binding in trans, which further increases the CFSE-CMRA neutrophil aggregation to ~19% without IL-8 and ~25% with IL-8.
